# Identification and Quantification of DNA Repair Protein Apurinic/Apyrimidinic Endonuclease 1 (APE1) in Human Cells by Liquid Chromatography/Isotope-Dilution Tandem Mass Spectrometry

**DOI:** 10.1371/journal.pone.0069894

**Published:** 2013-07-29

**Authors:** Güldal Kirkali, Pawel Jaruga, Prasad T. Reddy, Alessandro Tona, Bryant C. Nelson, Mengxia Li, David M. Wilson, Miral Dizdaroglu

**Affiliations:** 1 Biomolecular Measurement Division, National Institute of Standards and Technology, Gaithersburg, Maryland, United States of America; 2 Biosystems and Biomaterials Division, National Institute of Standards and Technology, Gaithersburg, Maryland, United States of America; 3 Laboratory of Molecular Gerontology, National Institute on Aging, National Institutes of Health, Baltimore, Maryland, United States of America; University of Massachusetts Medical School, United States of America

## Abstract

Unless repaired, DNA damage can drive mutagenesis or cell death. DNA repair proteins may therefore be used as biomarkers in disease etiology or therapeutic response prediction. Thus, the accurate determination of DNA repair protein expression and genotype is of fundamental importance. Among DNA repair proteins involved in base excision repair, apurinic/apyrimidinic endonuclease 1 (APE1) is the major endonuclease in mammals and plays important roles in transcriptional regulation and modulating stress responses. Here, we present a novel approach involving LC-MS/MS with isotope-dilution to positively identify and accurately quantify APE1 in human cells and mouse tissue. A completely ^15^N-labeled full-length human APE1 was produced and used as an internal standard. Fourteen tryptic peptides of both human APE1 (hAPE1) and ^15^N-labeled hAPE1 were identified following trypsin digestion. These peptides matched the theoretical peptides expected from trypsin digestion and provided a statistically significant protein score that would unequivocally identify hAPE1. Using the developed methodology, APE1 was positively identified and quantified in nuclear and cytoplasmic extracts of multiple human cell lines and mouse liver using selected-reaction monitoring of typical mass transitions of the tryptic peptides. We also show that the methodology can be applied to the identification of hAPE1 variants found in the human population. The results describe a novel approach for the accurate measurement of wild-type and variant forms of hAPE1 *in vivo*, and ultimately for defining the role of this protein in disease development and treatment responses.

## Introduction

A key pathway in protecting against the mutagenic and lethal consequences of many forms of DNA damage is base excision repair (BER) [1], [2]. BER copes with base modifications, apurinic/apyrimidinic (AP) sites, and both blocked ended (polymerase-non compatible) and clean single-strand breaks. The repair response is initiated by a DNA glycosylase, which excises a substrate base, leaving behind an AP site intermediate. While the AP site can be cleaved by multifunctional glycosylases, which harbor an intrinsic AP lyase activity, most abasic lesions are processed by apurinic/apyrimidinic endonuclease 1 (APE1). Following incision 5′ to the AP damage by APE1, DNA polymerase β removes the 5′-deoxyribose phosphate and replaces the missing nucleotide. In short-patch BER, the remaining nick is sealed by either DNA ligase 1 or a complex of XRCC1 and DNA ligase 3. In certain circumstances [3], BER proceeds via long-patch repair, which involves strand-displacement synthesis and engages the 5′-flap endonuclease FEN1. Defects in BER, and the related sub-pathway of DNA single-strand break repair, have been genetically linked to cancer predisposition, neurodegeneration and other diseases [4].

In addition to being the major AP endonuclease in mammals [5], APE1 plays important roles in transcriptional regulation and modulating stress responses. For instance, APE1 was purified based on its ability to stimulate the DNA binding activity of the AP-1 transcription factor complex [6]. Since then, APE1 has been shown to regulate the binding capacity of p53, NF-κB and HIF (among others) through both redox-dependent and -independent mechanisms [7]. APE1 also forms an integral part of a multiprotein transcriptional regulatory complex that operates to modulate gene expression, including of its own gene [8]. Moreover, APE1 is part of the SET complex, which is an endoplasmic reticulum-associated oxidative stress response complex that contains the nucleases NM23-H1 and TREX1 [9]. In addition, APE1 exhibits 3′ to 5′ exonuclease, 3′-phosphodiesterase and 3′-phosphatase activities on various non-conventional 3′ DNA termini [10], [11], and cleaves 5′ to certain oxidatively modified DNA bases to initiate “nucleotide incision repair” [12]. Finally, APE1 appears to play a role in removing damaged AP site-containing RNA molecules and in regulating the half-life of specific transcripts through site-specific mRNA cleavage [13].

Deletion of both alleles of *ape1* (or *Apex1*) in mice leads to early embryonic lethality, underscoring the critical nature of its associated functions [14]. In addition, APE1 heterozygosity in mice results in increased sensitivity to oxidative stress, reduced survival of pups and embryos, increased incidence of papillary adenocarcinoma and lymphoma, and elevated mutagenesis [15]–[17]. While sufficient depletion of APE1 in cells can lead to apoptosis [18], [19], inhibition or downregulation of this protein can sensitize cells to various DNA-damaging agents [20]. Moreover, defects in APE1 activity have been connected to loss of neuronal cell function and may contribute to the development of neurodegenerative disease [21]–[23].

Mounting evidence points to the predictive and prognostic value of APE1 expression and subcellular localization in human cancers [24], [25]. For instance, strict APE1 nuclear localization typically associates with a good prognosis, whereas combined cytoplasmic and nuclear localization correlates with poor survival. Increased APE1 expression, observed in multiple human cancer types, is also associated with resistance to chemotherapy and radiation therapy, and with poor survival in many cancers. Moreover, APE1 polymorphisms have been associated with cancer disposition [24], [25]. While a definitive association of APE1 dysfunction with a human disorder has not been reported, reduced-function APE1 proteins have been described that may represent susceptibility alleles [26].

In light of the discussion above, the accurate measurement of human APE1 (hAPE1) levels in tissues is necessary for evaluating hAPE1 as a predictive and prognostic biomarker in cancer and other diseases. In the past, hAPE1 expression levels have generally been estimated in clinical samples by semi-quantitative immunohistochemical methods. In this work, we describe the development of methodologies that use mass spectrometry with isotope-dilution that could permit the simultaneous positive identification, accurate quantification and genetic determination of hAPE1. Being able to reliably identify and quantify hAPE1 composition is a necessary step for assessing and predicting the involvement of this protein in disease etiology and therapeutic responsiveness.

## Materials and Methods

### Ethics Statement

Mouse livers were kindly provided by Dr. R. Stephen Lloyd (Oregon Health and Science University, Portland, Oregon), who stated the following: The breeding and care of the wild type C57BL/6J mice were in accordance with the protocols approved by the Animal Care and Use Committee of Oregon Health & Science University, Portland, Oregon (Protocol Number IS00002316 - A967). Prior to euthanasia by cervical dislocation, mice were anesthetized by CO_2_ inhalation. All efforts were made to minimize any discomfort to the mice, in accordance with approved animal care protocols.

### Materials

Trypsin (Proteomics Grade), acetonitrile (HPLC-grade) and water (HPLC-grade) for analysis by LC-MS/MS were purchased from Sigma (St. Louis, MO). Water purified through a Milli-Q system (Millipore, Bedford, MA) was used for all other applications. Acrylamide, bisacrylamide, and protease inhibitor cocktail tablets were obtained from Sigma-Aldrich (St. Louis, MO). Shodex carboxymethyl cellulose HPLC preparative column (CM 2025) was from Phenomenex (Torrance, CA). Diethylaminoethyl (DEAE) cellulose (DE52) was from Whatman laboratories (La Jolla, CA). YM10 membrane filters were from Millipore (Bedford, MA). ^15^N-NH_4_Cl was purchased from Cambridge Isotope Laboratories (Andover, MA). The Laemmli sample buffer, 12.5% Tris-HCl Criterion precast gels, 1xTris/Glycine/SDS running buffer and Coomassie Brilliant Blue R-250 Staining Solutions Kit were purchased from Bio-Rad Laboratories (Hercules, CA). The In-Gel Tryptic Digestion Kit was from Pierce (Rockford, IL). Prestained protein markers were from New England BioLabs (Ipswich, MA).

### Expression and Purification of hAPE1

Recombinant, untagged hAPE1 was expressed and purified from BL21 (λDE3) bacteria (Novagen) essentially as described [27]. In brief, following transformation of the pETApe recombinant plasmid, BL21 (λDE3) cells were grown at 37°C to an absorbance of ≈ 0.8 at OD_600_. IPTG was then added to a final concentration of 1 mM, and cells were grown overnight at 20°C. Bacteria were harvested, washed once with 1x phosphate-buffered saline (PBS) and lysed (after freezing) by sonication in 50 mM Hepes-KOH, pH 7.5, 50 mM KCl and 5% glycerol. The recombinant hAPE1 was immediately purified from the clarified extracts using sequential UNO Q12 and UNO S6 column (BioRad) chromatography as outlined previously [27]. The Q51H and G241R variant of hAPE1 were produced and purified as described [26].

### Expression and Purification of ^15^N-hAPE1

The relevant strains for ^15^N-hAPE1 production were *E. coli* Novablue (K12) and BL21 (λDE3). Minimal medium was prepared as described [28]. The composition of the medium was as follows: 6 g NaH_2_PO_4_, 3 g K_2_HPO_4_, 0.5 g NaCl, and 1 g ^15^N-NH_4_Cl, 5 g glucose, 246 mg MgSO_4_.7H_2_O per L. *E. coli* BL21 (λDE3) harboring pETApe recombinant plasmid was grown at 37°C for 20 h on LB agar plate containing 100 µg ampicillin/mL. A colony was carefully (without touching into the LB medium) transferred to 10 mL minimal medium containing 50 µg ampicillin/mL. Cells were grown for 3 h at 37°C at 250 rpm in a 50 mL tube. This inoculum was transferred to 200 mL minimal medium with 50 µg ampicillin/mL in a 500 mL flask. This culture was grown at 37°C for 14 h. Next, each 50 mL of this seed culture was transferred to 4×1000 mL of minimal medium containing ^15^N-NH_4_Cl and ampicillin in 4×2 L flasks. This culture was grown at 37°C for 6 h and then at 20°C for 90 min. Cell density at this stage was A_600_ = 0.5. The production of ^15^N-hAPE1 was induced with 100 µmol IPTG/mL at 20°C for 15 h. Cells were harvested at 6,000×g for 20 min and washed with 25 mM Tris buffer (pH 7.5). The wet weight of cells obtained in this procedure was 2.25 g/L culture for a total of 9 g wet weight of cells.

Nine grams of cells from 4 L culture were suspended in 90 mL of lysis buffer A: 50 mM HEPES-KOH buffer, pH 7.5, containing 5% glycerol, 50 mM KCl, and 1 mM DTT. Cells were broken by passing through a French Press at 7×10^4^ kPa. The cell-free extract was centrifuged at 70000×g for 1 h. The supernatant was mixed with 5 g of DE52 anion exchange resin equilibrated with the buffer A. The supernatant/resin slurry in a 250 ml bottle was mixed at 178 rpm for 1 h and then poured into a column. The flow through containing nearly all the hAPE1 and fewer cellular proteins was collected. The resin was washed with 10 mL of the buffer A and added to the flow through. The ^15^N-hAPE1 enriched pool (100 mL) was chromatographed on a HPLC-Shodex caboxymethyl cellulose column (2.0 cm×25 cm) equilibrated with buffer A. The column was washed with 100 mL of Buffer A until the A_280_ stabilized. Then, ^15^N hAPE1 was eluted with a potassium chloride gradient generated from Buffer A and Buffer A containing 0.65 M KCl (250 mL each). Pure ^15^N-hAPE1 was eluted as a sharp peak at ≈ 0.3 M KCl. Fractions containing ^15^N-hAPE1 were pooled and concentrated to ≈ 10 mg/mL on a YM10 membrane filter. The yield of ^15^N-hAPE1 from 4 L culture of minimal medium was 60 mg. Aliquots of the protein were stored at –70°C.

### Molecular Mass Determination of hAPE1 and^ 15^N-hAPE1 by Orbitrap Mass Spectrometry

Samples of each recombinant protein in storage buffer were dialyzed against Milli-Q water for 24 h, dried with a Speed Vac and reconstituted in 0.1% formic acid. Liquid chromatography/mass spectrometry (LC/MS) for the separation and accurate mass analysis of the proteins was conducted on a Thermo Scientific LTQ Orbitrap Discovery MS system (San Jose, CA) operated in positive ion mode, coupled to an Agilent 1200 HPLC system (Palo Alto, CA). For mass analysis, each respective protein sample was trapped onto a C8 guard column (2.1 mm diameter×1.25 cm length, Agilent) and eluted with a 9.5 min gradient operated at 200 µL/min flow rate. Solvent A was water containing 0.1% formic acid and solvent B was 80% acetonitrile +20% water containing 0.1% formic acid. The gradient settings were: 5% to 35% solvent B in 3 min, 35% to 70% solvent B in 5 min, 70% to 100% solvent B in 0.5 min, isocratic flow at 100% solvent B for 0.5 min and 5% solvent B in 0.5 min. Electrospray mass spectra were acquired over the range from *m/z* 150 to *m/z* 2000 during the chromatographic separations. The mass axis was externally calibrated with fluoroalkyl cyclophosphazene to assure accurate mass measurements. The following instrumental parameters were used for MS detection: spray voltage, 4 kV; sheath gas flow rate, 35 (arbitrary units); capillary temperature, 250°C; capillary voltage, 40 V; and tube lens, 200 V. Mass spectral deconvolution and protein molecular mass determination was performed using *ProMass* for Xcalibur, version 2.8 (Novatia Analytical Solutions, LLC; Monmouth Junction, NJ).

### AP Endonuclease Assays

AP endonuclease activity was measured essentially as described [29]. In brief, a 34-mer oligodeoxynucleotide (34F: CTGCAGCTGATGCGC**F**GTACGGATCCCCGGGTAC) harboring a single abasic site analogue (tetrahydrofuran or **F**) was 5′-[^32^P] end-labeled and annealed to a cold complementary strand (34G: GTACCCGGGGATCCGTACGGCGCATCAGCTGCAG). Incision reactions (final volume of 10 µL) consisted of 1 pmol of duplex DNA substrate and the designated amount of hAPE1 in 50 mM HEPES pH 7.5, 50 mM KCl, 1 mM MgCl_2_ and 1 mM DTT. Reactions were incubated at 37°C for 10 min, and stopped by the addition of an equal volume of 90% formamide/20 mM EDTA and heating at 95°C for 10 min. Following separation on a 15% polyacrylamide-urea denaturing gel, substrate and product bands were visualized and quantified on a Typoon Trio+ Variable Model Imager (Amersham Bioscience/GE Healthcare, Piscataway, NJ) using ImageQuant software (Molecular Dynamics, GE Healthcare).

### Cell Culture

Mammary gland epithelial adenocarcinoma cells (MCF7) (ATCC, Manassas, VA) were grown in Eagle′s Minimum Essential Medium (EMEM) (ATCC) containing 1.0 mM sodium pyruvate, 0.1 mM non-essential amino acids and 1.4 g/L sodium bicarbonate, and supplemented with penicillin (100 units/mL), streptomycin (100 µg/mL), 10% by volume fetal bovine serum (FBS ) (Invitrogen, Carlsbad, CA) and 0.01 mg/mL bovine insulin (Cell Applications, San Diego, CA). Mammary gland epithelial cells (MCF10A) (ATCC, Manassas, VA) were grown in Mammary Epithelium Growth Medium (MEGM) (Lonza, Walkersville, MD) supplemented with 100 ng/mL cholera toxin (Sigma, St. Louis, MO). Hepatocellular carcinoma cells (HepG2) (ATCC, Manassas, VA) were grown in EMEM supplemented with penicillin (100 units/mL), streptomycin (100 µg/mL) and 10% by volume FBS. All cells were maintained in a humidified 5% CO_2_ balanced-air atmosphere at 37°C.

### Extraction of Nuclear and Cytoplasmic Proteins from Human Cells and Mouse Liver

The extraction of nuclear and cytoplasmic proteins from cultured MCF7, MCF10A and HepG2 cells was performed using a commercially available kit (NE-PER, Thermo Scientific, Rockford, IL) according to the instructions of the manufacturer. Briefly, 3×10^6^ cells were washed twice by suspending the cell pellet in 1 mL PBS buffer. The cells were then transferred to an Eppendorf tube and centrifuged at 500×g for 5 min. The supernatant fraction was removed and discarded, leaving the cell pellet as dry as possible. An aliquot of 300 µL ice-cold Cytoplasmic Extraction Reagent I (CER I) was added to the pellet. The tube was vortexed on the highest setting for 15 s and then left on ice for 10 min. An aliquot of 16.5 µL of CER II was added to the tube, followed by vortexing for 5 s and then centrifuging for 5 min at 16000×g. The supernatant fraction containing the cytoplasmic extract was transferred to a pre-chilled tube. The pellet containing the nuclear extract was suspended in 150 µL ice-cold Nuclear Extraction Reagent. The sample was placed on ice and continuously vortexed on the highest setting for 15 s every 10 min, for a total of 40 min. The tube was then centrifuged at 16000×g for 10 min. The supernatant fraction was transferred to a pre-chilled tube and kept at –80°C until use.

For the protein extraction from mouse liver, 100 mg of dry tissue was cut into small pieces and washed twice with PBS buffer, and then centrifuged at 500×g for 5 min. The supernatant fraction was discarded so that the tissue pellet was as dry as possible. The tissue was homogenized using a Sonicator XL (Ultrasonic Processor) homogenizer in 1 mL of CER I. The subsequent steps were as described above for the protein extraction from cultured cells. Protein concentrations of the nuclear and cytoplasmic extracts from cultured cells and mouse tissue were determined using the Bradford method [30].

### Separation and Enrichment of APE1 from Nuclear and Cytoplasmic Extracts

In order to isolate and enrich APE1 prior to LC-MS/MS analysis, nuclear and cytoplasmic extracts were separated by HPLC using a liquid chromatograph equipped with an automatic injector, a diode-array detector, an automatic fraction collector (Agilent Technologies, Wilmington, DE) and a column specifically designed for protein separations (XBridge BEH300 C_4_, 4.6 mm×250 mm, 3.5 µm) with an attached guard column (Delta-Pak C4, 5 µm, 30 nm) (Waters, Milford, MA). Mobile phases A and B were water plus 0.1% TFA (v/v) and acetonitrile plus 0.1% TFA (v/v), respectively. A gradient starting from 20% B and linearly increasing to 72% B in 30 min was used. Afterwards, B was increased to 90% in 0.1 min and kept at this level for 5 min and then decreased to 20% to equilibrate the column for 25 min. The flow rate was 0.5 mL/min. The diode-array detector was used to monitor the effluents at 220 nm with reference to 360 nm. Prior to separation of protein extracts, an aliquot of hAPE1 was injected to determine its elution time range. Aliquots of nuclear and cytoplasmic protein extracts (100 µg and 250 µg, respectively) were spiked with an aliquot of ^15^N-hAPE1 as an internal standard and vortexed. Several 50 µL injections of each protein extract solution were performed with needle wash after each injection. The effluents corresponding to the elution time range (≈ 1.2 min) of hAPE1 were collected. The collected fractions were dried in a SpeedVac under vacuum prior to trypsin digestion. Protein extracts were also separated by SDS-PAGE as described previously [31]. Prestained protein standards, hAPE1 and ^15^N-hAPE1 were used as markers. The part of the gel corresponding to the migration time of hAPE1 was cut from the gel and divided into smaller pieces.

### Hydrolysis with Trypsin

An aliquot of 100 µg of hAPE1 or ^15^N-hAPE1 was incubated with 2 µg trypsin in 500 µL Tris-HCl buffer (30 mM, pH 8.0) at 37°C for 2 h. Then, an aliquot of 2 µg trypsin was added again. After another 22 h incubation, the sample was heated at 95°C for 5 min to deactivate trypsin prior to analysis by LC-MS/MS. Collected HPLC fractions were hydrolyzed in the same manner. In-gel tryptic digestion using the cut gel pieces was performed as described [31].

### Instrumentation and Analysis

LC-MS/MS analyses were performed on a Thermo-Scientific Accela High Speed LC system coupled to a Thermo-Scientific Finnigan TSQ Quantum Ultra AM triple quadrupole MS/MS system with an installed heated electrospray-ionization (HESI) source. Experimental conditions for the automated mass calibration and operating parameters of the MS/MS system in the positive ion mode were as previously described [32], except for sheath gas (nitrogen) pressure and scan width being 60 (arbitrary units) and *m/z* 2, respectively. Samples (40 µL) were analyzed using a Zorbax Extend-C-18, Rapid Resolution HT column (2.1 mm×100 mm, 1.8 µm particle size) (Agilent Technologies, Wilmington, DE) with an attached Agilent Eclipse XDB-C8 guard column (2.1 mm×12.5 mm, 5 µm particle size). The autosampler and column temperature were kept at 5°C and 40°C, respectively. Mobile phase A was water plus 2% acetonitrile and 0.1% formic acid (v/v), whereas mobile phase B consisted of acetonitrile plus 0.1% formic acid (v/v). A gradient analysis starting from 1% B and linearly increasing to 51% B in 25 min was used. Afterwards, B was increased to 90% in 0.1 min and kept at this level for 5 min and then decreased to 1% to equilibrate the column for 20 min. The flow rate was 300 µL/min.

### Statistical Analysis

Four independently prepared batches of the 3 cell lines, MCF-10A, MCF-7 and HepG2, and liver samples from 5 different mice were used to quantify APE1 levels. Statistical analyses of the data were performed using the GraphPad Prism 5.04 software (La Jolla, CA, USA) and two-tailed nonparametric Mann Whitney test with Gaussian approximation and confidence interval of 95%. A *p*-value <0.05 was assumed to correspond to statistical significance. More details are given in the Results section concerning the quantification of APE1 and the tryptic peptides used.

## Results

### Production, Purification and Characterization of Full Length ^15^N-labeled hAPE1

Both hAPE1 and ^15^N-hAPE1 were overexpressed in and purified from bacteria (Figure S1). Both recombinant proteins were analyzed by HPLC to check their purity and elution behavior. In each case, one single peak was observed with no discernible impurities (Figure S2). The retention times of both proteins were identical. The AP endonuclease activity of the purified ^15^N-hAPE1 was tested to ascertain its enzymatic efficiency using a 5′-[^32^P] end-labeled 34-mer oligodeoxynucleotide, which harbors a single abasic site analogue (tetrahydrofuran, **F**) and was annealed to a complementary strand. The results showed that the enzymatic activity of ^15^N-hAPE1 was essentially identical to that of hAPE1 (Figure S3), indicating no major perturbation of the active site by ^15^N-labeling or during subsequent purification.

### Mass Spectrometric Analysis of hAPE1 and ^15^N-hAPE1

Orbitrap mass spectrometry was used to measure the molecular masses of hAPE1 and ^15^N-hAPE1, and the isotopic purity of the latter. The calculated average molecular masses of hAPE1 and ^15^N-hAPE1 amount to 35554.6 Da and 35992.6 Da, respectively, assuming that all the N-atoms are labeled in ^15^N-hAPE1. The measured molecular masses hAPE1 and ^15^N-hAPE1 were 35422.0 Da and 35854.0 Da, respectively, indicating that an amino acid was missing from both molecules. It is well known that the N-terminal Met is excised from eukaryotic proteins by the actions of peptide deformylase and methionine aminopeptidase as an essential process, when the penultimate residue is small and uncharged such as Pro next to Met in hAPE1 [33]. Without the N-terminal Met, the calculated average molecular masses of hAPE1 and ^15^N-hAPE1 amounted to 35423.4 Da and 35860.4 Da, respectively. The measured molecular mass of hAPE1 differed from that of the theoretical value by 1.4 Da only, which is within the mass accuracy error of the Orbitrap mass spectrometer. In the case of ^15^N-hAPE1, the mass difference was 6.4 Da and the measured value amounted to 99.98% of the theoretical value. These data confirmed the identity of both hAPE1 and ^15^N-hAPE1, and suggested an almost complete labeling of hAPE1, making the labeled protein an excellent internal standard for the mass spectrometric measurement of hAPE1.

### Separation and Identification of Tryptic Peptides of hAPE1 and ^15^N-hAPE1

**Figure 1:**
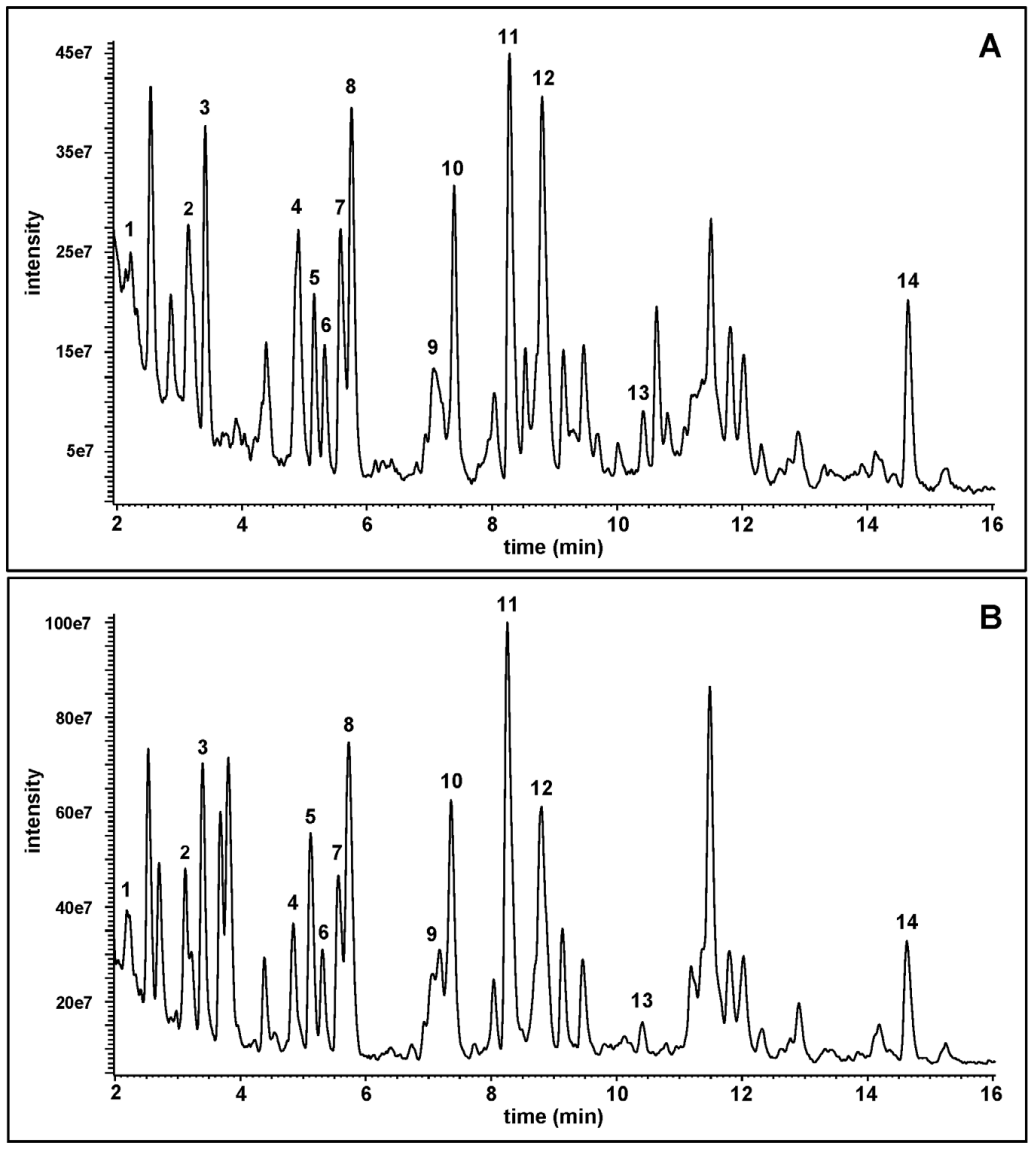
TIC profiles of the tryptic hydrolysates of hAPE1 (A) and ^15^N-hAPE1 (B). **The identities of the peptides are given in **Table 1****.****

To establish a signature proteolytic map of hAPE1, aliquots of unlabeled and ^15^N-labeled protein were hydrolyzed with trypsin. The hydrolysates were analyzed by LC-MS/MS to obtain the full-scan mass spectra of the separated tryptic peptides for identification. Trypsin hydrolyzes 44 peptide bonds in hAPE1, resulting in 1 arginine, 7 lysines and 37 peptides containing 2 to 22 amino acids (http://au.expasy.org/cgi-bin/peptide_cutter/peptidecutter.pl). In the total-ion-current (TIC) profile of the trypsin hydrolysate of hAPE1 (Figure 1A), 14 peptides that matched the theoretical tryptic peptides of hAPE1 were identified on the basis of their full-scan mass spectra. The trypsin hydrolysate of ^15^N-hAPE1 yielded an essentially identical TIC profile with 14 analogous peptides (Figure 1B). These 14 peptides covered a wide range in the hAPE1 sequence from the amino acid-18 (Gly) to amino acid-281 (Arg). Table 1 shows the identities of the tryptic peptides of both proteins and the monoisotopic masses of their protonated molecular ions (MH^+^) and doubly protonated (charged) molecular ions [(M+2H)^2+^]. Fourteen tryptic peptides yielded a protein score of 150 with the use of the “MASCOT” search engine (http://www.matrixscience.com); protein scores greater than 56 are considered significant (p<0.05) for positive identification. Furthermore, using the SwissProt database (http://prospector.ucsf.edu/prospector/cgi-bin/mssearch.cgi) and just four tryptic peptides (e.g., NAGFTPQER, GAVAEDGDELR, EGYSGVGLLSR and QGFGELLQAVPLADSFR), which cover 15.1% of the protein (48 of the 318 amino acids), the MS-Fit Search with taxonomy search *Homo Sapiens* selecting 20479 entries in the database resulted in a 100% match with hAPE1. Thus, the simultaneous measurement of just those four tryptic peptides is sufficient to positively identify and quantify hAPE1 in human cells.

**Table 1:** Identification of the tryptic peptides in Figure 1 and the *m/z* values (Th) of the monoisotopic masses of their MH^+^ and (M+2H)^2+^ ions.

		unlabeled		^15^N-labeled	
peak	peptide	MH^+^	(M+2H)^2+^	MH^+^	(M+2H)^2+^
1	GLASR	503.29	252.14	511.29	256.14
2	TSPSGKPATLK	1086.64	543.82	1099.64	550.32
3	GLVR	444.29	222.64	452.29	226.64
4	NAGFTPQER	1019.49	510.24	1033.49	517.24
5	NVGWR	631.33	316.16	641.33	321.16
6	GAVAEDGDELR	1131.53	566.26	1145.53	573.26
7	VSYGIGDEEHDQEGR	1690.73	845.86	1711.73	856.36
8	AWIK	517.31	259.15	523.31	262.15
9	EAAGEGPALYEDPPDQK	1786.82	893.90	1805.82	903.40
10	WDEAFR	823.37	412.18	833.37	417.18
11	GLDWVK	717.39	359.19	725.39	363.19
12	EGYSGVGLLSR	1137.59	569.29	1151.59	576.29
13	EEAPDILCLQETK	1488.73	744.86	1503.73	752.36
14	QGFGELLQAVPLADSFR	1847.96	924.48	1869.96	935.48

### Full-scan Mass Spectra of the Tryptic Peptides

**Figure 2:**
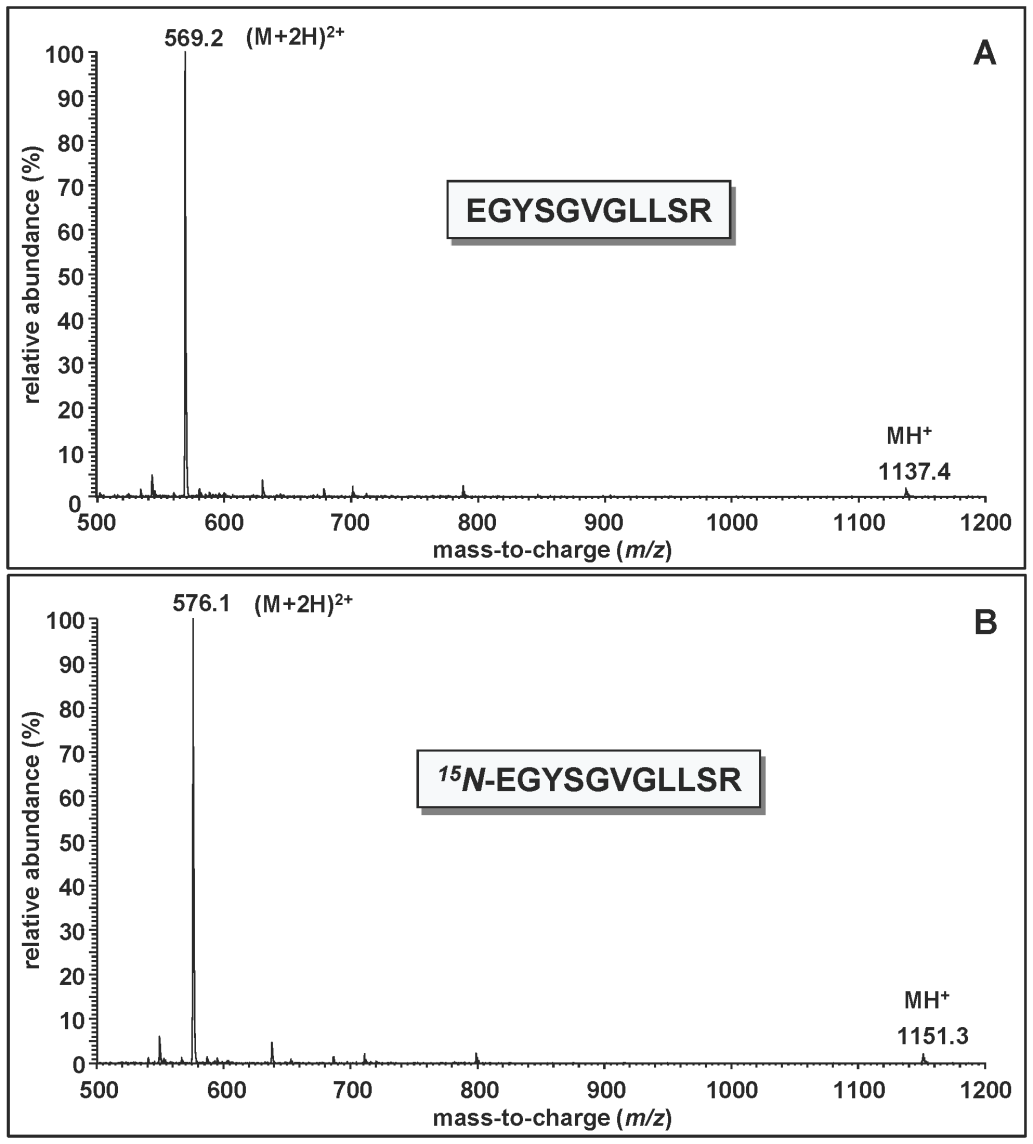
Full-scan mass spectra of EGYSGVGLLSR (A) (represented by peak 12 in Figure 1A) and ^15^N-EGYSGVGLLSR (B) (represented by peak 12 in Figure 1B).

An (M+2H)^2+^ ion and an MH^+^ ion of lower abundance were observed in the full-scan spectra of all fourteen tryptic peptides of both proteins. For example, the full-scan mass spectrum of the peptide EGYSGVGLLSR (represented by peak 12 in Figure 1A) contained an (M+2H)^2+^ ion as the base peak at *m/z* 569 and an MH^+^ ion at *m/z* 1137 (Figure 2A). The full-scan mass spectrum of the ^15^N-labeled analogue of this peptide (represented by peak 12 in Figure 1B) exhibited a shift of 14 Da in the mass of MH^+^ (Figure 2B), consistent with the fourteen ^15^N atoms in the molecule. Other examples of the full-scan spectra of the tryptic peptides are shown in Figures S4–S7. No masses of unlabeled material were observed in the spectra of the ^15^N-labeled peptides, consistent with the molecular mass measurement of ^15^N-hAPE1 (see above).

### Production Spectra of the Tryptic Peptides

**Figure 3:**
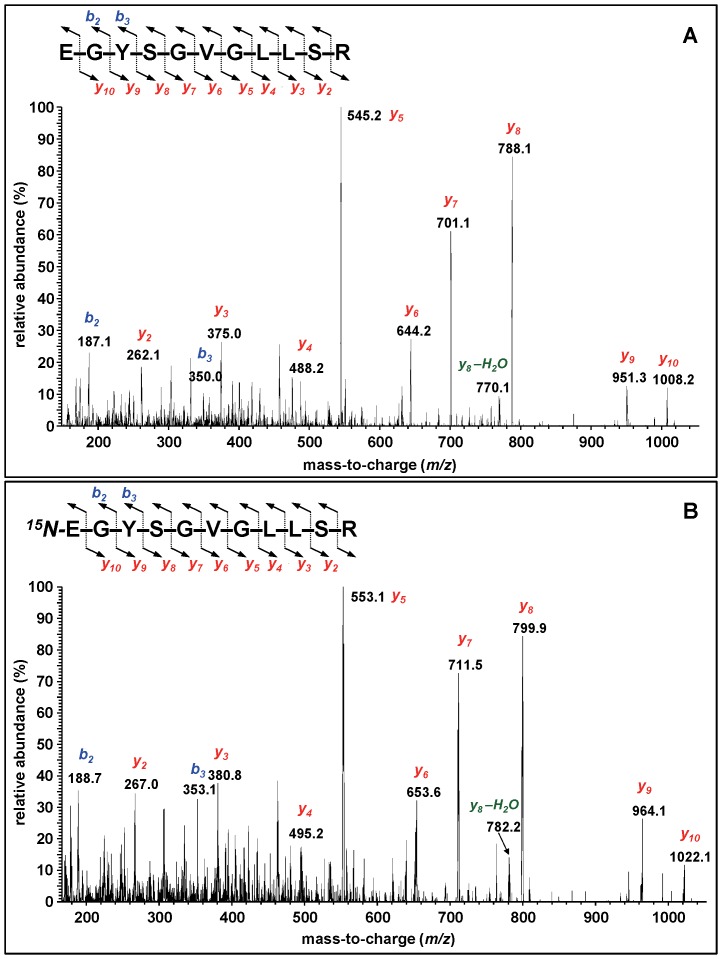
Product ion spectra of EGYSGVGLLSR (A) and ^15^N-EGYSGVGLLSR (B). The (M+2H)^2+^ ions *m/z* 569.3 and *m/z* 576.3 (Table 1), respectively, were used as the precursor ions.

We calculated the theoretical masses of the typical *y*- and *b*-series ions as the product ions [34] that are expected to result from the collision-induced fragmentation of the identified tryptic peptides. As an example, Table S1 shows the calculation of the masses of the *y*- and *b*-ions of EGYSGVGLLSR and ^15^N-EGYSGVGLLSR. The calculated masses of the theoretical *y*- and *b*-ions of the identified tryptic peptides of hAPE1 and ^15^N-hAPE1 are given in Tables S2 and S3, respectively. The optimum collision energy to obtain a product ion spectrum was chosen according to the empirically and experimentally determined collision energies for (M+2H)^2+^ ions as the precursor ions [31], [34]. As an example, Figure 3A illustrates the product ion spectrum of EGYSGVGLLSR, which exhibited the typical *y*-ion series from the *y_2_*-ion to the *y_10_*-ion, with the most intense ion being the *y_5_*-ion at *m/z* 545. Only a few *b*-ions were observed. ^15^N-EGYSGVGLLSR gave an essentially identical product ion spectrum with mass shifts according to the ^15^N-content of the fragments (Figure 3B **,** Tables S2 and S3). Product ion spectra of the other tryptic peptides and their ^15^N-labeled analogues were also dominated by the *y*-ions, whereas some *b*-ions were discernible with lower abundance. Figures S8-S11 illustrate other examples of product ion spectra. Ions resulting by loss of NH_3_ or H_2_O from *y-* or *b-*ions were observed in some cases.

### Selected-reaction Monitoring

To check the suitability of LC-MS/MS for the identification and quantification of hAPE1 at low concentrations, selected-reaction monitoring (SRM) was used to analyze the trypsin hydrolysate of a mixture of hAPE1 and ^15^N-hAPE1. (M+2H)^2+^ was chosen as the precursor ion for transitions, because this ion generally represents the highest charge state of tryptic peptides [34], and because it was more prominent than MH^+^ (Figure 2, Figures S4–S7). Ion-current profiles of the mass transitions of tryptic peptides of hAPE1 and ^15^N-hAPE1 exhibited excellent peak shapes and a base-line separation between the peptides (Figure S12). Each peptide and its respective ^15^N-labeled analogue co-eluted at the same retention time. To further ascertain the identity of a target peptide, multiple typical transitions can be simultaneously monitored [35]. For example, the case of EGYSGVGLLSR and QGFGELLQAVPLADSFR demonstrates that the ion-current profiles of their five typical transitions lined up at the appropriate retention time for each peptide, confirming their identity (Figure S13).

The analytical sensitivity of the instrument was measured using SRM and a number of tryptic peptides of hAPE1 and the transition from (M+2H)^2+^ to the most intense ion in the product ion spectrum of each peptide. Depending on the peptide, the limit of detection (LOD) varied from approximately 10 fmol to 20 fmol, with a signal-to-noise ratio (S/N) of at least 3. The limit of quantification (LOQ) was approximately 50 fmol to 100 fmol of target peptide with an S/N of 10. The MS/MS response to increasing concentration ratios of the tryptic peptides and their ^15^N-labeled analogues was assumed to be linear within the ratio limits that we previously reported for tryptic peptides of proteins with similar lengths, hNEIL1 and hOGG1 [31], [32].

### Identification of APE1 in Human Cells and Mouse Liver

**Figure 4:**
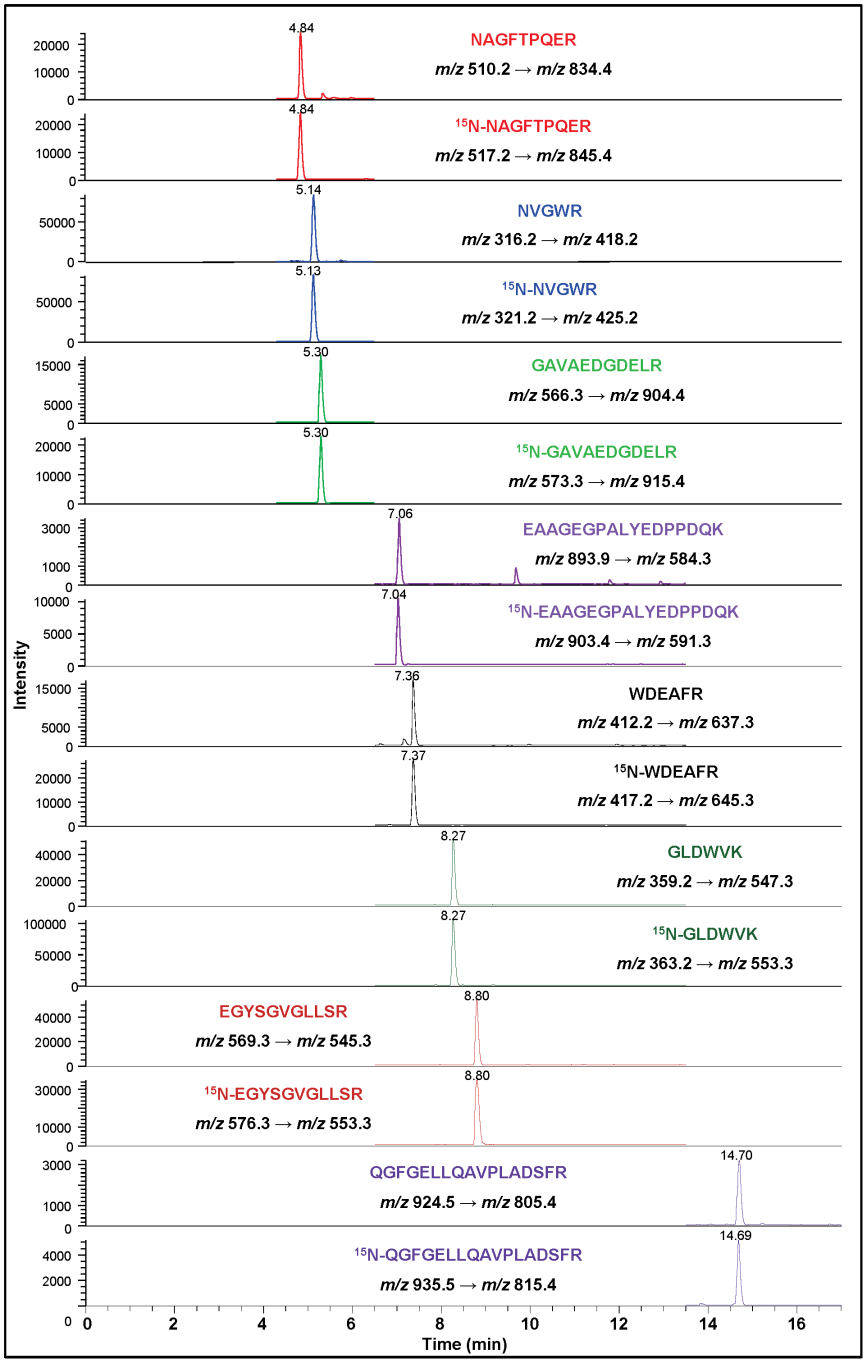
Ion-current profiles of mass transitions of eight tryptic peptides of hAPE1 and ^15^N-hAPE1 obtained using the tryptic hydrolysate of a protein fraction, which was collected during separation by HPLC of a nuclear extract of MCF-10A cells. The extract was spiked with an aliquot of ^15^N-hAPE1 prior to separation. Peptides and monitored transitions are shown.

Having established the basic methodology, we attempted to identify APE1 in three cultured human cell lines: MCF-10A (mammary gland epithelial cell line), MCF-7 (mammary gland epithelial adenocarcinoma cell line) and HepG2 (hepatocellular carcinoma cell line). Nuclear and cytoplasmic extracts were prepared, in part to look at intracellular distribution patterns. To enrich hAPE1 for LC-MS/MS analysis, aliquots of the extracts were spiked with an aliquot of ^15^N-hAPE1 and then separated by HPLC (Figure S14). The fractions, which corresponded to the elution time and baseline width of hAPE1, were collected. The collected fractions were lyophilized, hydrolyzed with trypsin and analyzed by LC-MS/MS with SRM of typical mass transitions of the tryptic peptides of both hAPE1 and ^15^N-hAPE1. As Figure 4 shows, the signals of the eight typical mass transitions of hAPE1 were observed at the expected retention times together with those of the corresponding mass transitions of ^15^N-hAPE1, unequivocally identifying hAPE1 initially in MCF-10A cells. This procedure was repeated for each of the other two cell lines using both nuclear and cytoplasmic extracts with similar results. As an example, Figure S15 illustrates the identification of hAPE1 in MCF-7 cells.

**Figure 5:**
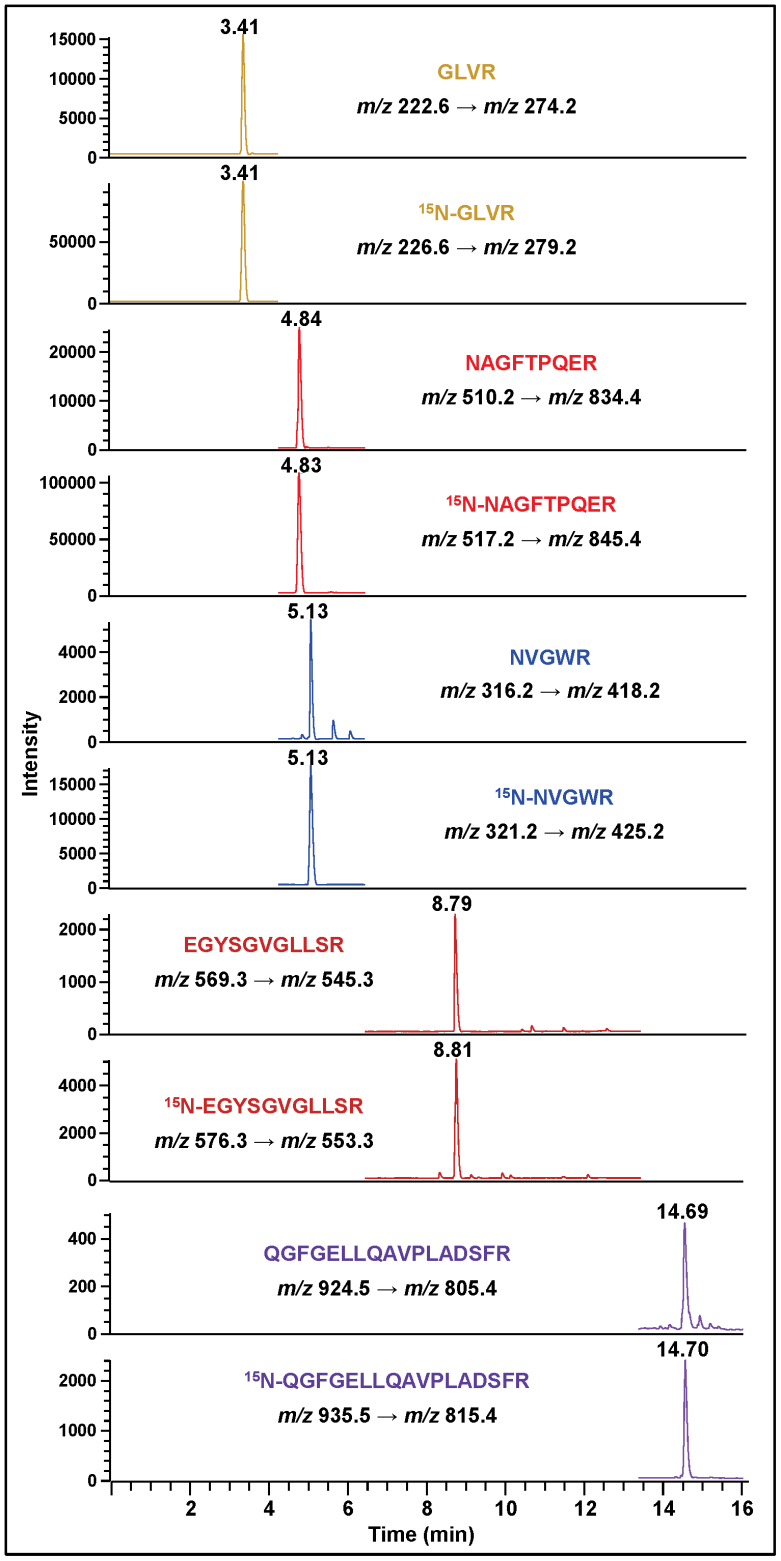
Ion-current profiles of mass transitions of five tryptic peptides of hAPE1 and ^15^N-hAPE1 obtained using the in-gel tryptic hydrolysate of protein bands, which were excised from the gel following the separation of nuclear extracts of HepG2 cells by SDS-PAGE (Figure S12). Aliquots of nuclear extracts were spiked with an aliquot of ^15^N-hAPE1 prior to SDS-PAGE. Peptides and monitored transitions are shown.

As an alternative strategy, sodium dodecyl sulfate-polyacrylamide gel electrophoresis (SDS-PAGE) was used to separate the extracts and subsequently isolate hAPE1 for analysis by LC-MS/MS (Figure S16). Prior to separation, aliquots of the extracts were spiked with an aliquot of ^15^N-hAPE1. Regions of the gel were excised that corresponded to the migration position of hAPE1 and subjected to in-gel tryptic hydrolysis [31]. As Figure 5 illustrates, LC-MS/MS analysis of the tryptic hydrolysates clearly identified hAPE1 in HepG2 cell extracts using the distinguishable ion-current profiles of typical mass transitions.

**Figure 6:**
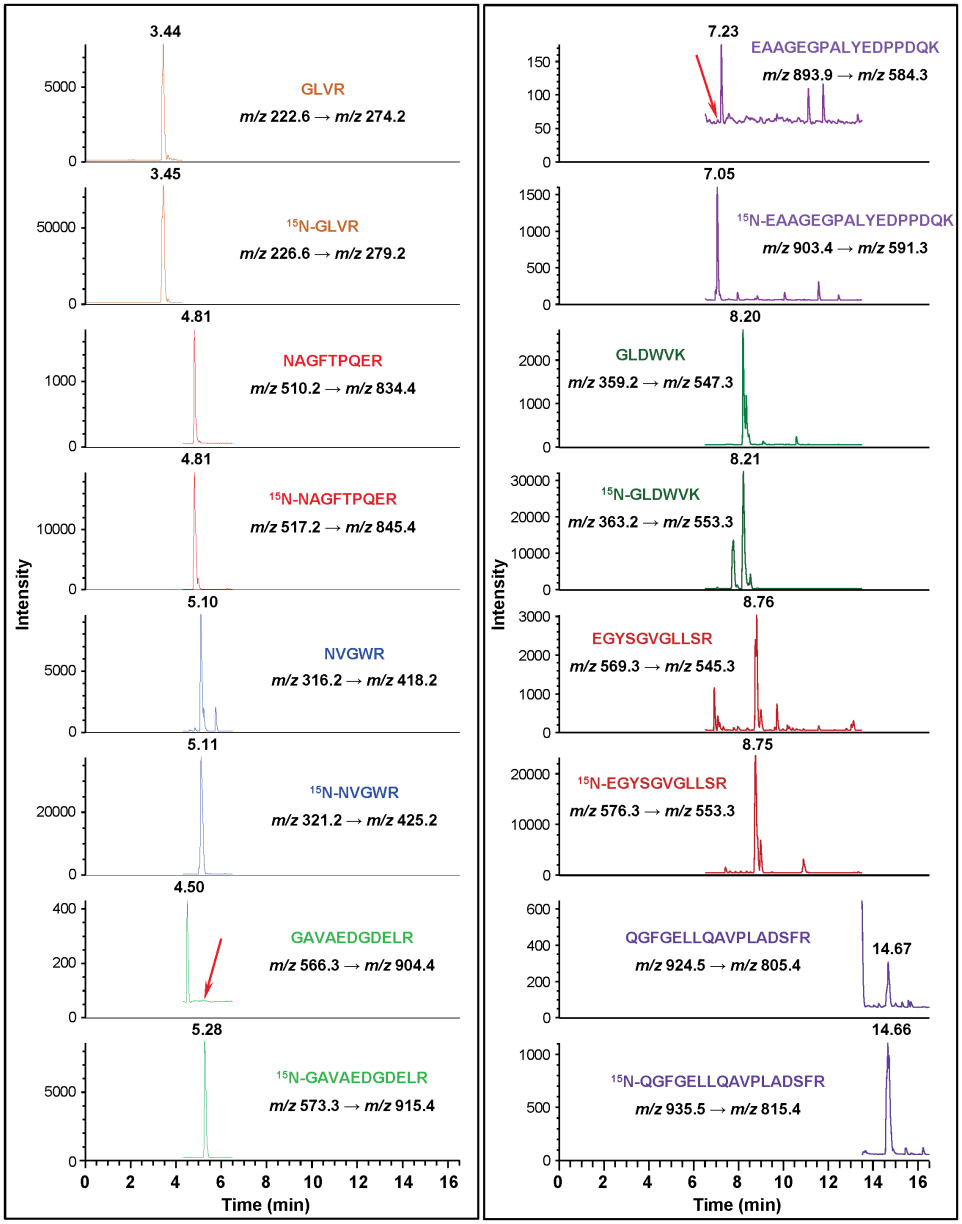
Ion-current profiles of mass transitions of eight tryptic peptides of hAPE1 and ^15^N-hAPE1 obtained using the tryptic hydrolysate of a protein fraction, which was collected during separation by HPLC of a nuclear extract of mouse liver. The nuclear extract was spiked with an aliquot of ^15^N-hAPE1 prior to HPLC-separation. Peptides and monitored transitions are shown. The red arrows indicate the elution positions of GAVAEDGDELR and EAAGEGPALYEDPPDQK, which are absent, because they are not among the tryptic peptides of mAPE1.

We next attempted to identify APE1 in mouse liver to test the suitability of our approach for measuring APE1 in tissues. Human tissues could not be used at the present time because of the difficulties in obtaining material transfer agreements. Eight of the fourteen identified tryptic peptides of hAPE1 (indicated in Table 1 by the numbers 3, 4, 5, 8, 10, 11, 12 and 14) are identical to eight theoretical tryptic peptides of mouse APE1 (mAPE1) (http://au.expasy.org/cgi-bin/peptide_cutter/peptidecutter.pl). Nuclear and cytoplasmic extracts were isolated from mouse liver, spiked with a known amount of ^15^N-hAPE1 and separated by HPLC as described above. Since hAPE1 (318 amino acids) and mAPE1 (317 amino acids) have 94% similarity in amino acid sequence, close average molecular masses (35555 Da vs. 35491 Da), and nearly identical chemical and physical properties [36], we assumed that mAPE1 would have similar retention behavior as hAPE1. Thus, proteins eluting with a retention time that corresponded to hAPE1 were collected, hydrolyzed with trypsin and analyzed by LC-MS/MS with SRM. Mass transitions of the eight tryptic peptides were monitored. As Figure 6 illustrates, six of the tryptic peptides, whose amino acid sequences are identical in both hAPE1 and mAPE1, were observed at the expected retention times together with those of the mass transitions of their corresponding^ 15^N-labeled analogues, unequivocally identifying APE1 in mouse liver. Signals of the mass transitions of the two tryptic peptides GAVAEDGDELR and EAAGEGPALYEDPPDQK were clearly absent as indicated by red arrows in Figure 6, because the sequence of the former peptide does not exist in mAPE1 and the latter contains a Val instead of an Ala at position-42, which shifts the *m/z* value of (M+2H)^2+^ from 893.9 Th to 907.9 Th.

### Quantification of APE1 in Human Cells and Mouse Liver

**Figure 7:**
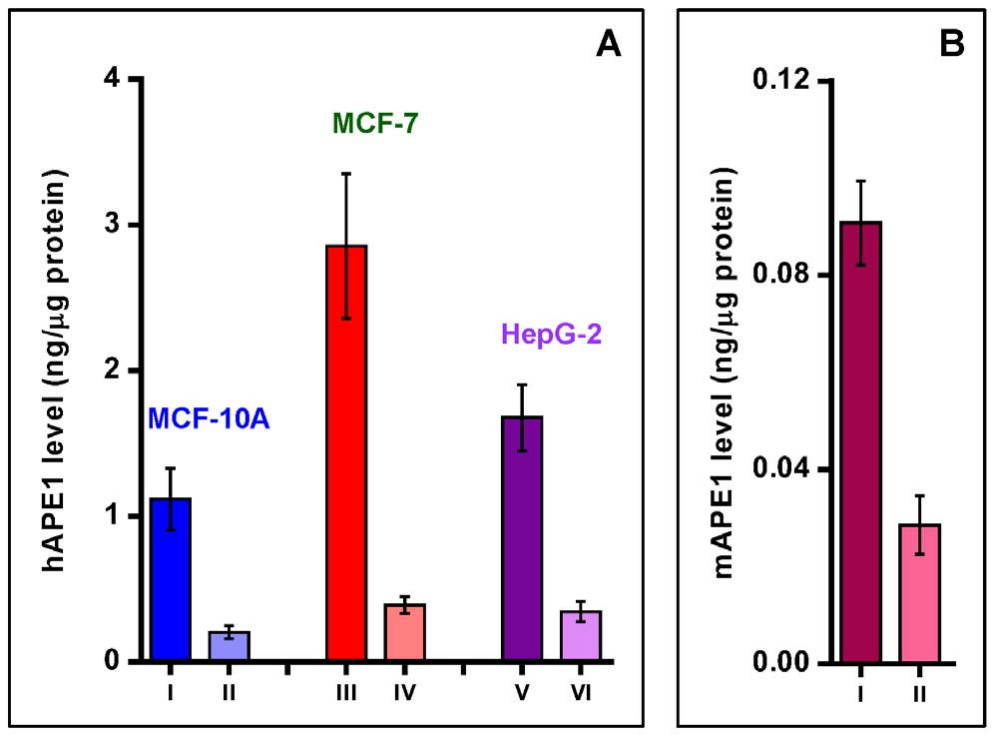
Levels of APE1 (ng/µg protein) in nuclear extracts (I, III and V) and cytoplasmic extracts (II, IV and VI) of MCF-10A, MCF-7 and HepG2 cells (A), and in nuclear extract (I) and cytoplasmic extract (II) of mouse liver (B). The uncertainties are standard deviations. Statistical analysis: **A**: I vs. II, *p* = 0.0015; III vs. IV and V vs. VI, *p* < 0.0001; I vs. III and III vs. V, *p* < 0.0001; I vs. V, *p* = 0.026; II vs. IV, *p* < 0.0001; II vs. VI, *p* = 0.035. There is no statistical difference between IV and VI. **B**: I vs. II, *p* < 0.0001.

Four independently prepared batches of MCF-10A, MCF-7 and HepG2 cells, and the livers from five different mice were used to quantify APE1 levels. Using the basic procedures outlined above for hAPE1, the typical mass transitions of eight peptides (GLVR, NAGFTPQER, GAVAEDGDELR, EAAGEGPALYEDPPDQK, WDEAFR, GLDWVK, EGYSGVGLLSR and QGFGELLQAVPLADSFR) from each sample and their ^15^N-labeled analogues were monitored by SRM. For the reasons mentioned earlier, transitions of six peptides were used for determining the level of mAPE1 in each liver sample. The level of APE1 was calculated for each tryptic peptide using the measured signals of mass transitions of these peptides and their ^15^N-labeled analogues, the amount of the internal standard, and the protein amount in each extract. The means of four independent measurements of each peptide were combined to calculate the mean level of APE1 and the uncertainty of the measurements in cultured human cells and in mouse liver. Figure 7 illustrates the levels of APE1 in nuclear and cytoplasmic extracts of the three human cell lines and mouse liver. The statistical analysis of the data is given in the figure legend. In all cases, significantly greater levels of APE1 were found in nuclear extracts than in cytoplasmic extracts. The greatest level of APE1 was observed in MCF-7 cells. The level of APE1 in HepG2 cells was also significantly greater than in MCF-10A cells. The cytoplasmic extracts of MCF-7 and HepG-2 cells exhibited a similar level of APE1. However, this level was significantly greater than that in the cytoplasmic extract of MCF-10A cells. Mouse liver exhibited the lowest level of APE1.

### Detection of hAPE1 Variants

**Figure 8:**
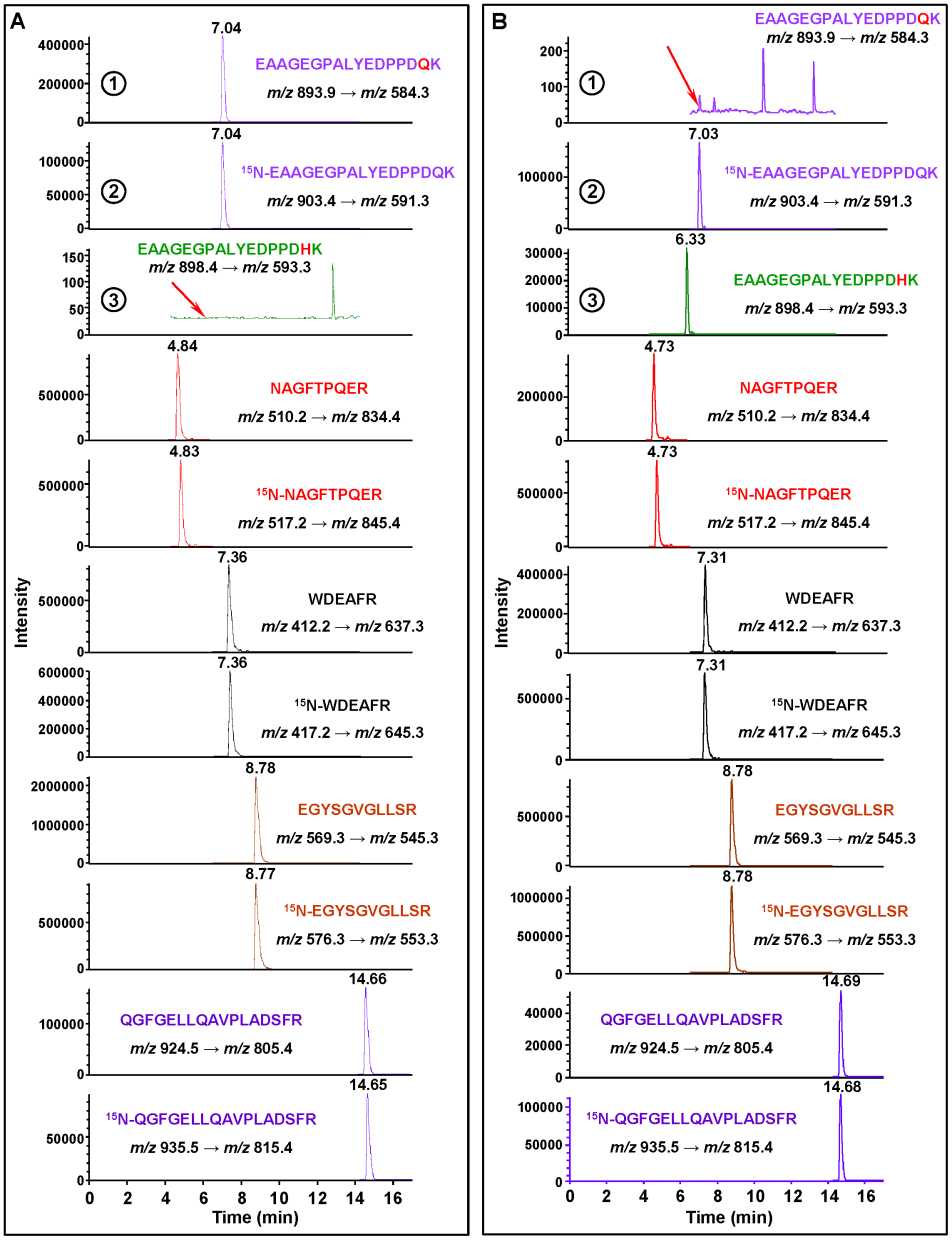
Detection of the Gln51His variant of hAPE1. **A:** wild type hAPE1, **B:** Gln51His variant. Ion-current profiles of the mass transitions of EAAGEGPALYEDPPD**Q**K of wild type hAPE1 (**1**), ^15^N-EAAGEGPALYEDPPDQK of ^15^N-hAPE1 (the internal standard) (**2**) and EAAGEGPALYEDPPD**H**K of Gln51His variant (**3**). The elution position of EAAGEGPALYEDPPD**H**K, which is absent in wild type hAPE1 (**A3**) and that of EAAGEGPALYEDPPD**Q**K, which is absent in Gln51His variant (**B1**), are indicated by the red arrows. Ion-current profiles of the mass transitions of four other tryptic peptides that are common to both wild type hAPE1 and Gln51His variant are also shown along with those of the corresponding ^15^N-labeled analogues.

We tested the applicability of the developed methodology to the detection of APE1 variants with single amino acid replacements. We overexpressed and purified two variants, Gln51His and Gly241Arg, as described previously [26], [27]. The missense polymorphism Gln51His occurs with a 0.048 frequency in endometrial and ovarian cancer patients combined [37], and at a 0.03 frequency in the general population [38]. Gln51 is located in the N-terminal portion of APE1, which is required for its redox function [39], [40]. The Gln51His variant may have functional significance because it represents the replacement of a non-conservative amino acid [37]. Gln51 is located in the tryptic peptide EAAGEGPALYEDPPD**Q**K identified in this work (Table 1). The substitution of Gln51 with His changes the *m/z* values of MH^+^ (1786.8 Th) and (M+2H)^2+^ (893.9 Th) of this peptide to 1795.8 Th and 898.4 Th, respectively. The masses of the *y_2_*- to *y_16_*-ions are also changed as calculated according to the example shown in Table S1. Among *b*-series ions, however, only the mass of the *b_16_*-ion is changed. The Gln51His variant was hydrolyzed with trypsin and then analyzed by LC-MS/MS. The tryptic peptide EAAGEGPALYEDPPD**H**K of this variant was identified by its full-scan mass spectrum that contained MH^+^ and (M+2H)^2+^ at *m/z* 1795.8 and *m/z* 898.4, respectively, and by its product ion spectrum with the *y_5_*-ion at *m/z* 593.3 as the most intense ion and with other expected *y*-series ions (data not shown). Next, aliquots of wild type hAPE1 and the Gln51His variant were spiked with an aliquot of ^15^N-hAPE1, hydrolyzed with trypsin and analyzed by LC-MS/MS with SRM. The typical mass transitions from (M+2H)^2+^ to the *y_5_*-ion of EAAGEGPALYEDPPD**Q**K and EAAGEGPALYEDPPD**H**K were recorded. Figure 8 illustrates the ion-current profiles of these transitions (**A1**, **A3**, **B1** and **B3**) and the corresponding transition of ^15^N-EAAGEGPALYEDPPDQK (internal standard) (**A2** and **B2**). As examples, the mass transitions of four other tryptic peptides that are common to both proteins are also shown, along with those of the corresponding ^15^N-labeled analogues. Figure 8A shows the presence of the transition of EAAGEGPALYEDPPD**Q**K of wild type hAPE1 (**A1**) and the absence of the transition of EAAGEGPALYEDPPD**H**K of the Gln51His variant (**A3**). In contrast, Figure 8B clearly shows the absence of the transition of EAAGEGPALYEDPPD**Q**K (**B1**) at its retention time (indicated by a red arrow) and the presence of the transition of EAAGEGPALYEDPPD**H**K (**B3**). The substitution of Gln by His apparently caused a shift in the retention time (Figure 8B3) to an earlier time.

Gly241 is located in the nuclease domain of APE1 [39]. The Gly241Arg variant occurs with an allele frequency of 0.004 in the general population and represents a non-conservative substitution [26]. The tryptic peptide QGF**G**ELLQAVPLADSFR identified in this work (Table 1) contains Gly241. When Gly is substituted by Arg, the resulting peptide QGF**R**ELLQAVPLADSFR would produce two peptides QGF**R** and ELLQAVPLADSFR, due to cleavage of the peptide bond next to Arg upon trypsin hydrolysis. This means that QGF**G**ELLQAVPLADSFR of wild type hAPE1 and QGF**R**ELLQAVPLADSFR are expected to be absent in the trypsin hydrolysate of the Gly241Arg variant. To identify the resulting peptides QGF**R** and ELLQAVPLADSFR, this variant was hydrolyzed with trypsin and analyzed by LC-MS/MS. Both peptides were identified by their full-scan mass spectra that contained MH^+^ and (M+2H)^2+^ at *m/z* 507.3 and *m/z* 254.1 (QGF**R**), and at *m/z* 1458.8 and *m/z* 729.9 (ELLQAVPLADSFR) (data not shown). Further evidence for the presence of these peptides was provided by their product ion ion spectra (Figures S17A,B), containing typical *y*-series ions with the *y_3_*-ion at *m/z* 379.2 (QGF**R**) and the *y_7_*-ion at *m/z* 805.3 (ELLQAVPLADSFR) being the most intense ions. Next, an aliquot of the Gly241Arg variant was spiked with an aliquot of ^15^N-hAPE1, hydrolyzed with trypsin and analyzed by LC-MS/MS with SRM. The typical mass transitions from (M+2H)^2+^ to the *y_7_*-ion of QGF**G**ELLQAVPLADSFR and ^15^N-QGFGELLQAVPLADSFR were recorded along with two mass transitions of QGF**R** and four mass transitions of ELLQAVPLADSFR (Figure S18). As expected, the signal of the mass transition of QGF**G**ELLQAVPLADSFR was absent at its retention time (Figure S18-1) (indicated by a red arrow) in contrast to that of ^15^N-QGFGELLQAVPLADSFR (Figure S18-2). The signals of two transitions from (M+2H)^2+^ to *y_2_*- and *y_3_*-ions of QGFR (Figures S18-3 and S18-4) and of four transitions from (M+2H)^2+^ to *y_7_*-, *y_8_*-, *y_9_*- and *y_10_*-ions of ELLQAVPLADSFR (Figures S18-5 to S18-8) were observed at the retention times known from the previous analysis to obtain the full-scan spectra. All other identified tryptic peptides of wild type hAPE1 (Table 1) were found in the trypsin hydrolysate of the Gly214Arg variant (data not shown). Taken together, these results clearly demonstrate the applicability of the developed methodology to the identification of variants of hAPE1.

## Discussion

APE1 is a ubiquitous and multifunctional protein with critical roles in BER and other DNA damage processing pathways [5], [10], [41], and plays an important role in dictating DNA repair capacity, which can determine individual disease susceptibility and patient responsiveness to clinically-relevant DNA-damaging agents [42]. Knowledge of the expression levels and genetic composition of hAPE1 has the potential to provide a greater understanding of the DNA repair capacity of a given tissue and in guiding life style practices or treatment strategies in cancer. We describe herein a novel approach for the positive identification and absolute quantification of APE1 in human cells. The applicability of this approach to mammalian tissues and in the detection of hAPE1 variants was also demonstrated using mouse livers and purified recombinant proteins, respectively.

Unlike techniques that use isotopically labeled tryptic peptides, we used full length ^15^N-labeled hAPE1 as an internal standard for absolute quantification. This type of an internal marker, which possesses identical chemical and physical properties to the target protein, can be spiked into a sample at the earliest step of sample preparation. This attribute would compensate for eventual losses during all stages of analysis and prevent measurement bias due to trypsin digestion that can often be inefficient depending on the measured protein. Moreover, the use of a full length ^15^N-labeled internal standard is required for enrichment of the target protein during HPLC or one- or two-dimensional SDS-PAGE fractionation. The measurement of the molecular mass of ^15^N-hAPE1 by Orbitrap mass spectrometry showed a high degree of ^15^N-labeling of the molecule. This finding was confirmed by full-scan mass and product ion spectra of tryptic peptides. The absence of unlabeled material in ^15^N-hAPE1 met an absolute requirement for an ideal stable isotope-labeled internal standard. Moreover, ^15^N-hAPE1 exhibited identical properties to unlabeled hAPE1, including AP endonuclease activity, proving its suitability as an internal standard.

We achieved the positive identification and absolute quantification of hAPE1 in cultured human cells and of mAPE1 in mouse tissue using the developed methodology. We anticipate that in the future this approach can be applied to primary human cells or tissue biopsies for appropriate clinical investigations. The most common method to measure APE1 in human specimens is currently immunohistochemistry. While this strategy is comparatively easy to execute and can provide a quick assessment of APE1 protein intracellular distribution, it is strictly qualitative and completely dependent on a reliable and specific antibody resource. We demonstrate herein the ability to quantitatively measure the levels of APE1 in three different human cell lines and mouse liver, using both nuclear and cytoplasmic extracts. Consistent with what we know about this protein, nuclear extracts showed greater levels of APE1 than cytoplasmic extracts [25], [43]. The overall greater level of APE1 in MCF-7 cells than in MCF-10A cells is on a par with the increased APE1 expression previously observed in cancer cells relative to normal cells [24]. Moreover, the observed variability in the nuclear:cytoplasmic APE1 level ratio may reflect altered subcellular localization often seen in cancer cells [24], [25], [43]. The much lower level of APE1 observed in mouse liver relative to the human cell lines is striking, and may indicate effects of cell culturing on APE1 expression.

A real strength of the methodology described within is that it not only permits the accurate, quantitative measurement of APE1 *in vivo*, but would also allow determination of the overall APE1 content (i.e., the *APE1* genotype). A number of variants of hAPE1 have been identified in the human population and in a few cases of endometrial cancer, and these sequence alterations have the potential to lead to differences in repair capacity through either change in protein activity or expression level [26], [37], [38]. For instance, variants Leu104Arg, Asp148Glu, Gly241Arg, Glu126Asp, Arg237Ala, and Gly306Ala have been cloned, expressed, purified and characterized biochemically. The variants Leu104Arg, Glu126Asp and Arg237Ala exhibited ≈ 40% to 60% reductions in endonuclease activity, whereas the activities of the Gly241Arg and Gly306Ala variants were similar to that of wild type APE1 [26]. The Asp148Glu variant is the most common missense mutant with an allele frequency of ≈ 0.38 [26], [37], [38]. Although the recessive allele has been suggested to be involved in cancer disposition, such as for melanoma [44]–[46], and in sporadic amyotrophic lateral sclerosis (ALS) [47], this variant possesses normal AP endonuclease repair activity [26], raising doubt about its role in these diseases.

We demonstrated within the ability of the developed methodology to identify single amino acid substitutions in hAPE1 by analyzing two variants, Gln51His and Gly241Arg. The identity of the Gln51His variant was demonstrated by the absence of the wild type tryptic peptide EAAGEGPALYEDPPD**Q**K and the presence of the tryptic peptide EAAGEGPALYEDPPD**H**K with His at position-51. The Gly241Arg variant was identified by the absence of the wild type tryptic peptide QGF**G**ELLQAVPLADSFR and the appearance of the peptides QGF**R** and ELLQAVPLADSFR that resulted from tryptic cleavage of the bond next to Arg at position-241 (QGF**R**ELLQAVPLADSFR) in this variant. Another example of the ability of the methodology to detect amino acid differences among APE1 molecules was the distinction of mAPE1 from hAPE1 via the absence of two tryptic peptides.

We note that many other hAPE1 variants would be readily identified by an altered tryptic peptide profile, allowing for direct profiling of APE1 composition. For instance, in the case of the most common missense variant Asp148Glu, the replacement of Asp with Glu would change the *m/z* values of MH^+^ and (M+2H)^2+^ ions of the identified tryptic peptide VSYGIGDEEHDQEGR that contains Asp148. The masses of *y_9_*- to *y_14_*-ions and *b_7_*- to *b_14_*-ions would also be changed accordingly. These alterations would be identified by SRM of the corresponding mass transitions, thus permitting easy identification of this polymorphic variant and rapid genotyping for the 148 allele. As for the Glu126Asp variant, which has been found in an ALS patient [47], [48], the substitution of Glu126 by Asp in the tryptic peptide EGYSGVGLLSR would change the *m/z* values of the MH^+^, (M+2H)^2+^ and *b_1_*- to *b_10_*-ions, but not affect the *m/z* values of the *y*-ions, which would be readily identified by SRM of appropriate mass transitions. In the case of the Arg237Ala and Arg237Cys variants, the latter of which has been identified in endometrial cancer [26], [37], [38], Arg237 is located in the identified tryptic peptide NAGFTPQER. Since substitution of Arg by Ala or Cys removes a trypsin cleavage site, this peptide would not be generated and a larger (theoretical) peptide [NAGFTPQEAL**A**(or **C)**QGFGELLQAVPLADSFR] would result. The disappearance of NAGFTPQER would readily be detected, and the large peptide would likely be identified, revealing the identity of this variant.

In conclusion, we have described, for the first time, a novel approach with the use of LC-MS/MS for identifying and quantifying a human DNA repair protein in cell and tissue extracts, and for identifying variants of hAPE1. Future experiments will need to explore its broader utility and capacity to identify known and novel hAPE1 variants using patient samples. It should be noted that a sophisticated instrument such as a tandem mass spectrometer connected to a liquid chromatograph was used for the studies described in the present work. We believe such instruments can routinely be used for the measurement of APE1 as shown here and of other proteins as many laboratories around the world including our laboratory demonstrated in the past.

## Supporting Information

Figure S1 **Purification steps of ^15^N-hAPE1. SDS-PAGE analysis.** Lane 1: Uninduced cell extract (24 µg), Lane 2: Induced cell extract (28 µg), Lane 3∶70,000×g Supernatant fraction (22 µg), Lane 4: Flow through from DEAE cellulose (17 µg), Lane 5: Flow through from CM cellulose (15 µg), Lane 6: Purified ^15^N-hAPE1 (21 µg), Lane 7: Molecular mass markers from top to bottom in kDa **–** 175, 80, 58, 46, 30 and 25.
(TIFF)

Figure S2 **Separation of hAPE1 and ^15^N -hAPE1 by HPLC. The elution profiles were superimposed.**
(TIFF)

Figure S3 **Characterization of human ^15^N-hAPE1. Left:** hAPE1 and ^15^N-hAPE1 (1 µg each) were separated on a SDS-PAGE and visualized by Coomassie blue R250 staining. Each purified recombinant protein appears as a single band, with the molecular mass standards from top to bottom in kDa **–** 250, 150, 100, 75, 50, 37, 25 and 20. **Right:** hAPE1 and ^15^N-hAPE1 were assayed for AP endonuclease activity, with the incision reaction products being separated on a 15% polyacrylamide-urea denaturing gel. Assays were performed with 50, 100, 300 or 500 pg of hAPE1 and ^15^N-hAPE1 or with no enzyme. The 5′-^32^P-end labeled 34F substrate and the 18-mer incision product are indicated. The specific AP endonuclease activities of hAPE1 and ^15^N-hAPE1 were 0.17 ± 0.026 pmol (of substrate converted to product)·min^−1^·ng^−1^ and 0.11±0.025 pmol· min^−1^·ng^−1^, respectively.
(TIFF)

Figure S4 **Full-scan mass spectra of the tryptic peptides NAGFTPQER (A) and ^15^N-NAGFTPQER (B).**
(TIFF)

Figure S5 **Full-scan mass spectra of the tryptic peptides GAVAEDGDELR (A) and ^15^N-GAVAEDGDELR (B).**
(TIFF)

Figure S6 **Full-scan mass spectra of the tryptic peptides WDEAFR (A) and ^15^N-WDEAFR (B).**
(TIFF)

Figure S7 **Full-scan mass spectra of the tryptic peptides QGFGELLQAVPLADSFR (A) and ^15^N-QGFGELLQAVPLADSFR (B).**
(TIFF)

Figure S8 **Product ion spectra of the tryptic peptides NAGFTPQER (A) and ^15^N-NAGFTPQER (B).** The (M+2H)^2+^ ions *m/z* 510.2 (**A**) and *m/z* 517.2 (**B**) were used as the precursor ions. The insert shows the fragmentation pathways leading to the *b*- and *y*-ions.
(TIFF)

Figure S9 **Product ion spectra of the tryptic peptides GAVAEDGDELR (A) and ^15^N-GAVAEDGDELR (B).** The (M+2H)^2+^ ions *m/z* 566.3 (**A**) and *m/z* 573.3 (**B**) were used as the precursor ions. The insert shows the fragmentation pathways leading to the *b*- and *y*-ions.
(TIFF)

Figure S10 **Product ion spectra of the tryptic peptides WDEAFR (A) and ^15^N-WDEAFR (B).** The (M+2H)^2+^ ions *m/z* 412.2 (**A**) and *m/z* 417.2 (**B**) were used as the precursor ions. The insert shows the fragmentation pathways leading to the *b*- and *y*-ions.
(TIFF)

Figure S11 **Product ion spectra of the tryptic peptides QGFGELLQAVPLADSFR (A) and ^15^N-QGFGELLQAVPLADSFR (B).** The (M+2H)^2+^ ions *m/z* 924.5 (**A**) and *m/z* 935.5 (**B**) were used as the precursor ions. The insert shows the fragmentation pathways leading to the *b*- and *y*-ions.
(TIFF)

Figure S12 **Ion-current profiles of typical mass transitions of seven tryptic peptides of hAPE1 and ^15^N-hAPE1.** A tryptic digest of a mixture of hAPE1 and ^15^N-hAPE1 was used.
(TIFF)

Figure S13 **Ion-current profiles of five mass transitions of the tryptic peptides EGYSGVGLLSR (A) and QGFGELLQAVPLADSFR (B). A tryptic digest of a mixture of hAPE1 and ^15^N-hAPE1 was used.**
(TIFF)

Figure S14 **Separation by HPLC of a nuclear extract (A) and a cytoplasmic extract (B) of MCF-7 cells.** The elution profile of ^15^N-hAPE1 (**Fig. S2**) was superimposed to show the retention time period where collections were made to enrich hAPE1 for analysis by LC-MS/MS.
(TIFF)

Figure S15 **Ion-current profiles of mass transitions of eight tryptic peptides of hAPE1 and ^15^N-hAPE1 obtained using the tryptic digest of a fraction, which was collected during separation by HPLC of a nuclear extract of MCF-7 cells.** The extract was spiked with an aliquot of ^15^N-hAPE1 prior to separation by HPLC.
(TIFF)

Figure S16 **Separation of nuclear extracts of HepG2 cells by SDS-PAGE.** **1:** Molecular mass markers from top to bottom in kDa –175, 80, 58, 46, 30, 25 and 17; **2:** hAPE1; **3∶** ^15^N-hAPE1; **4–12:** nuclear extracts of HepG2 cells.
(TIFF)

Figure S17 **Product ion spectra of QGFR (A) and ELLQAVPLADSFR (B).**
(TIFF)

Figure S18 **Detection of the Gly241Arg variant of hAPE1.** Ion-current profiles of the mass transitions of QGFGELLQAVPLADSFR (**1**) and its ^15^N-labeled analogue (**2**), and two mass transitions of QGF**R**, (**3, 4**) and four mass transitions of ELLQAVPLADSFR (**5–8**). The elution position of QGFGELLQAVPLADSFR, which is absent in the Gly241Arg variant, is shown by the red arrow.
(TIFF)

Table S1 **Calculation of the masses (Da) of the** ***y*** **- and** ***b*** **-ions of EGYSGVGLLSR and ^15^N-EGYSGVGLLSR.** The first row shows the number of the ^15^N atoms in each amino acid.
(TIFF)

Table S2 **The masses (Da) of the theoretical** ***y*** **-series ions of the identified tryptic peptides of hAPE1 and their ^15^N-labeled analogues.**
(TIFF)

Table S3 **The masses (Da) of the theoretical** ***b*** **-series ions of the identified tryptic peptides of hAPE1 and their ^15^N-labeled analogues.**
(TIF)
