# Supplementary figures and images for: Identification and Quantification of DNA Repair Protein Apurinic/Apyrimidinic Endonuclease 1 (APE1) in Human Cells by Liquid Chromatography/Isotope-Dilution Tandem Mass Spectrometry
Source: PLoS One. 2013 Jul 29;8(7):e69894. doi: 10.1371/journal.pone.0069894 (PMC3726725; doi:10.1371/journal.pone.0069894)

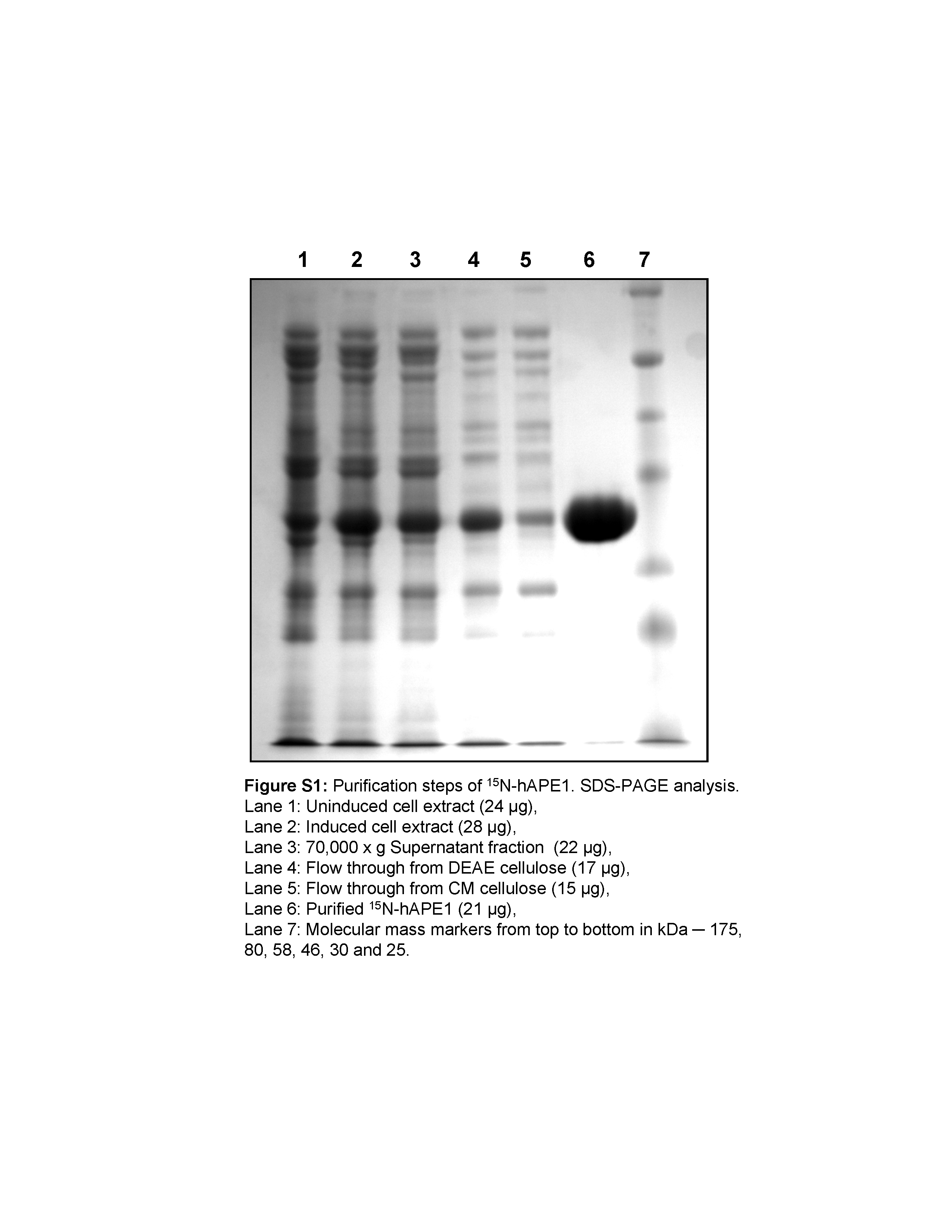

Supplement: Figure S1 — Purification steps of 15N-hAPE1. SDS-PAGE analysis. Lane 1: Uninduced cell extract (24 µg), Lane 2: Induced cell extract (28 µg), Lane 3∶70,000×g Supernatant fraction (22 µg), Lane 4: Flow through from DEAE cellulose (17 µg), Lane 5: Flow through from CM cellulose (15 µg), Lane 6: Purified 15N-hAPE1 (21 µg), Lane 7: Molecular mass markers from top to bottom in kDa – 175, 80, 58, 46, 30 and 25. (TIFF) [file pone.0069894.s001.tiff]

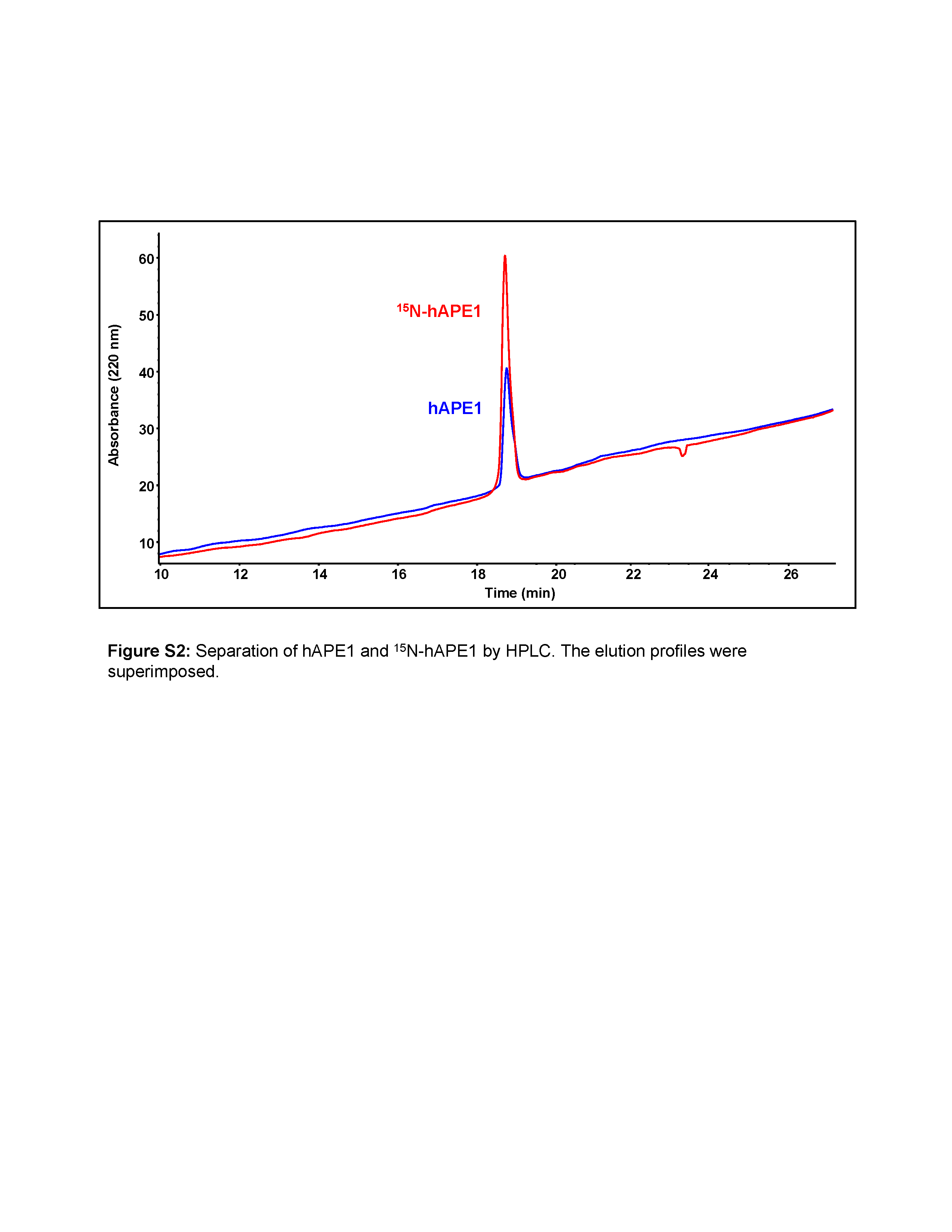

Supplement: Figure S2 — Separation of hAPE1 and 15N -hAPE1 by HPLC. The elution profiles were superimposed. (TIFF) [file pone.0069894.s002.tiff]

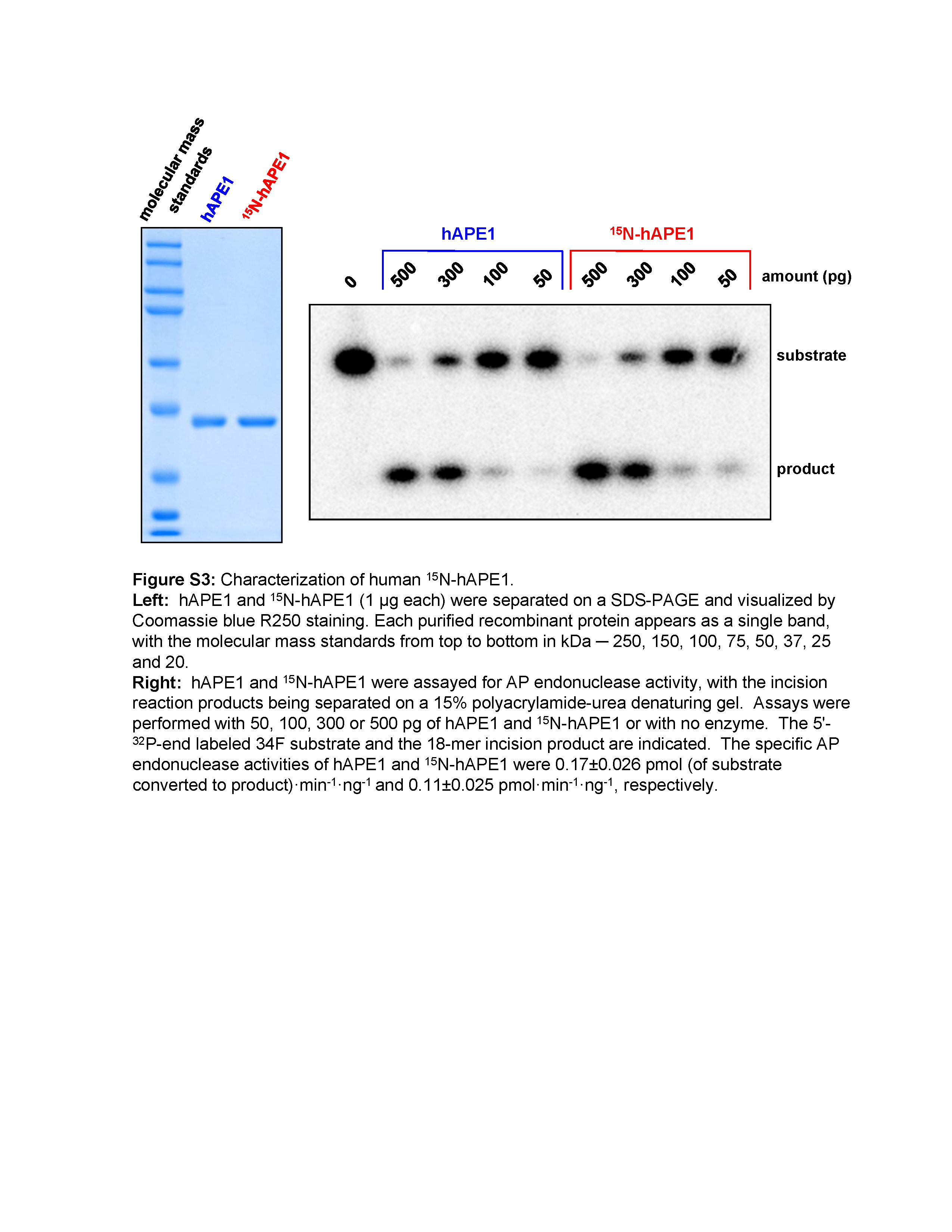

Supplement: Figure S3 — Characterization of human 15N-hAPE1. Left: hAPE1 and 15N-hAPE1 (1 µg each) were separated on a SDS-PAGE and visualized by Coomassie blue R250 staining. Each purified recombinant protein appears as a single band, with the molecular mass standards from top to bottom in kDa – 250, 150, 100, 75, 50, 37, 25 and 20. Right: hAPE1 and 15N-hAPE1 were assayed for AP endonuclease activity, with the incision reaction products being separated on a 15% polyacrylamide-urea denaturing gel. Assays were performed with 50, 100, 300 or 500 pg of hAPE1 and 15N-hAPE1 or with no enzyme. The 5′-32P-end labeled 34F substrate and the 18-mer incision product are indicated. The specific AP endonuclease activities of hAPE1 and 15N-hAPE1 were 0.17 ± 0.026 pmol (of substrate converted to product)·min−1·ng−1 and 0.11±0.025 pmol· min−1·ng−1, respectively. (TIFF) [file pone.0069894.s003.tiff]

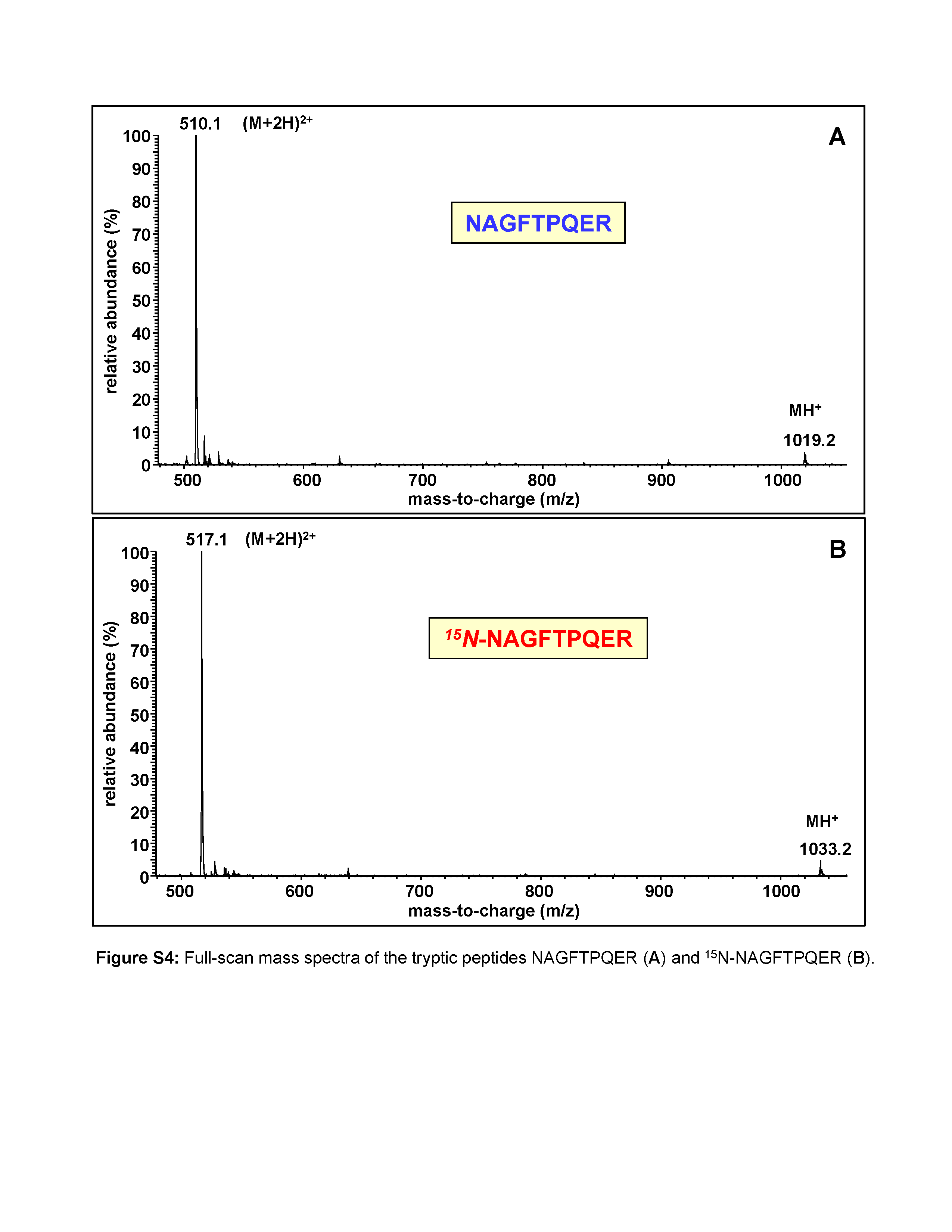

Supplement: Figure S4 — Full-scan mass spectra of the tryptic peptides NAGFTPQER (A) and 15N-NAGFTPQER (B). (TIFF) [file pone.0069894.s004.tiff]

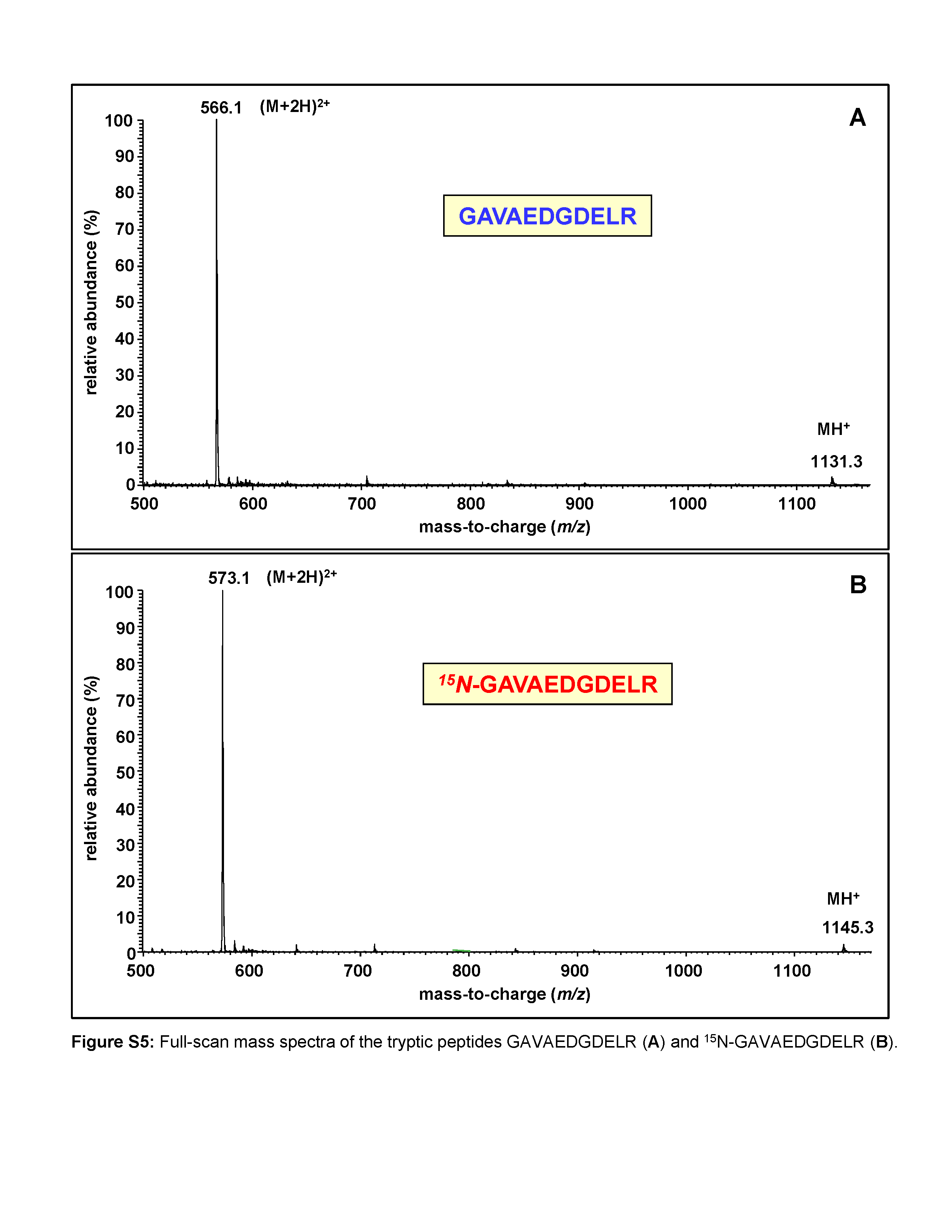

Supplement: Figure S5 — Full-scan mass spectra of the tryptic peptides GAVAEDGDELR (A) and 15N-GAVAEDGDELR (B). (TIFF) [file pone.0069894.s005.tiff]

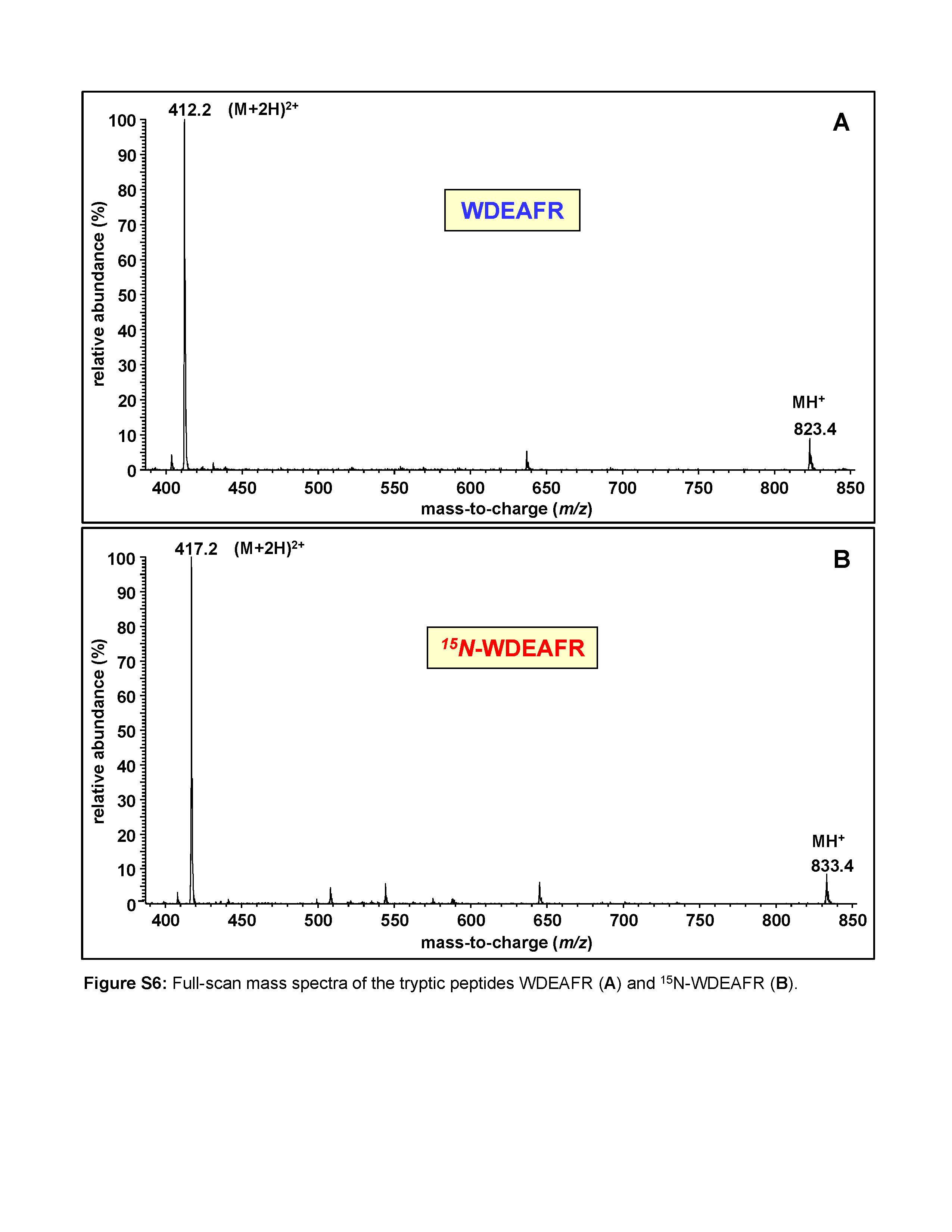

Supplement: Figure S6 — Full-scan mass spectra of the tryptic peptides WDEAFR (A) and 15N-WDEAFR (B). (TIFF) [file pone.0069894.s006.tiff]

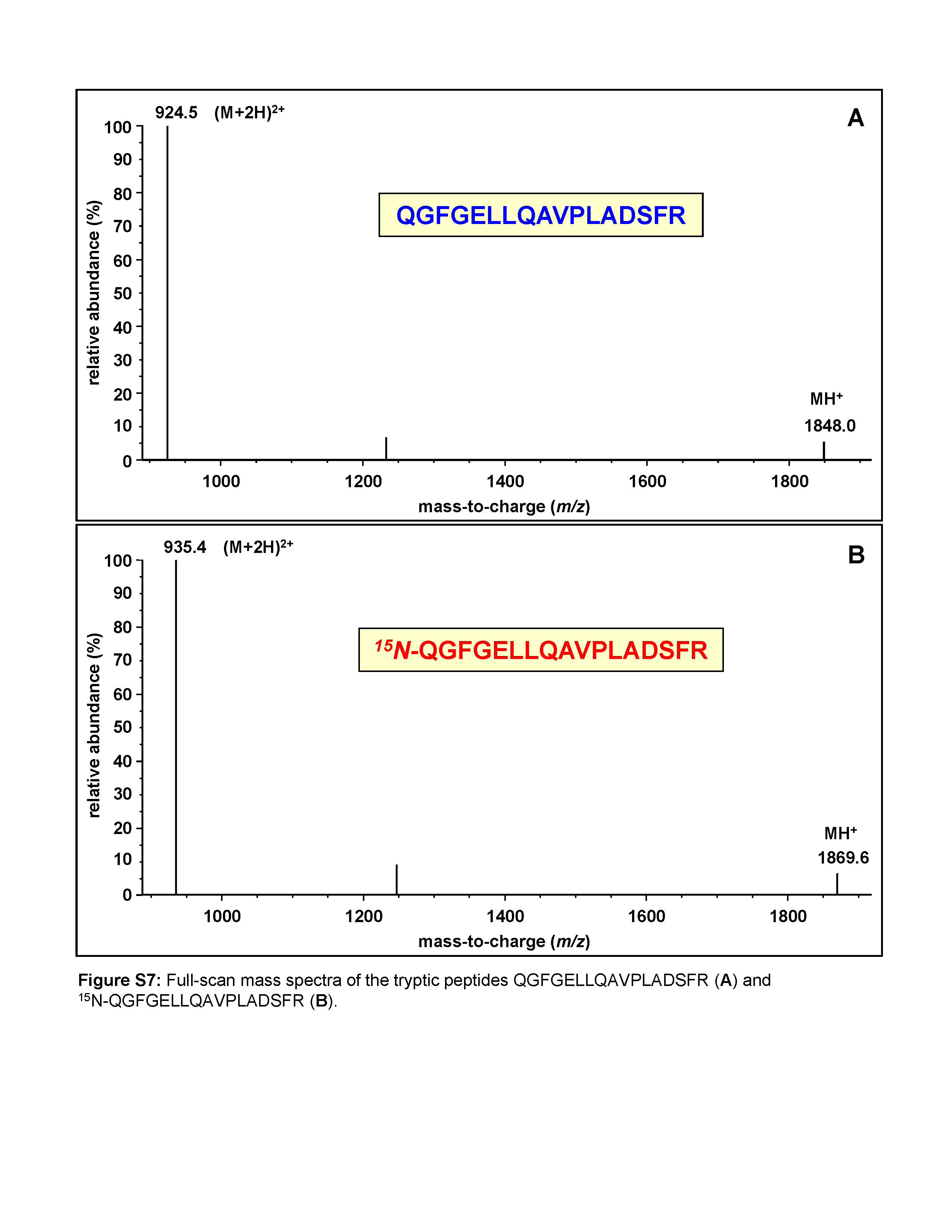

Supplement: Figure S7 — Full-scan mass spectra of the tryptic peptides QGFGELLQAVPLADSFR (A) and 15N-QGFGELLQAVPLADSFR (B). (TIFF) [file pone.0069894.s007.tiff]

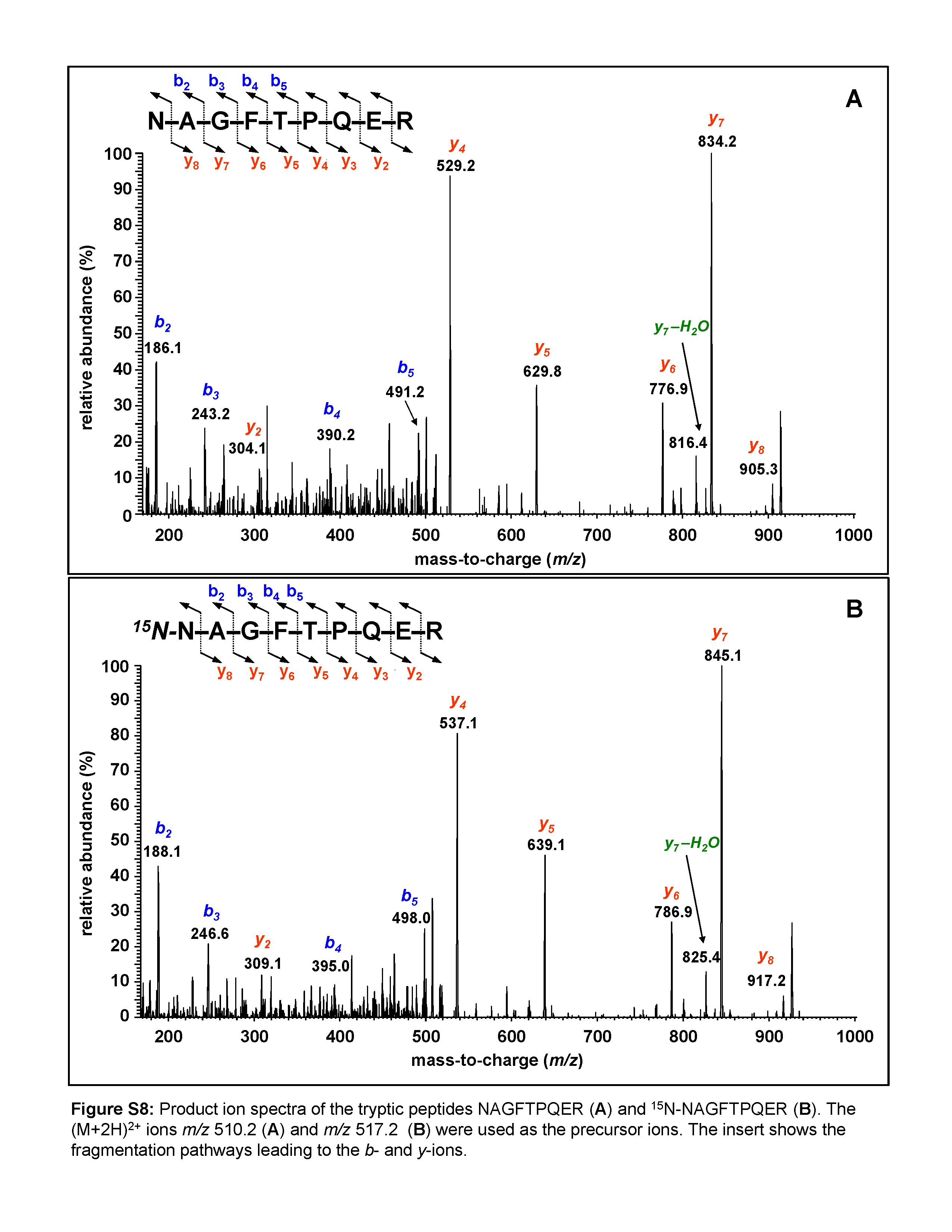

Supplement: Figure S8 — Product ion spectra of the tryptic peptides NAGFTPQER (A) and 15N-NAGFTPQER (B). The (M+2H)2+ ions m/z 510.2 (A) and m/z 517.2 (B) were used as the precursor ions. The insert shows the fragmentation pathways leading to the b- and y-ions. (TIFF) [file pone.0069894.s008.tiff]

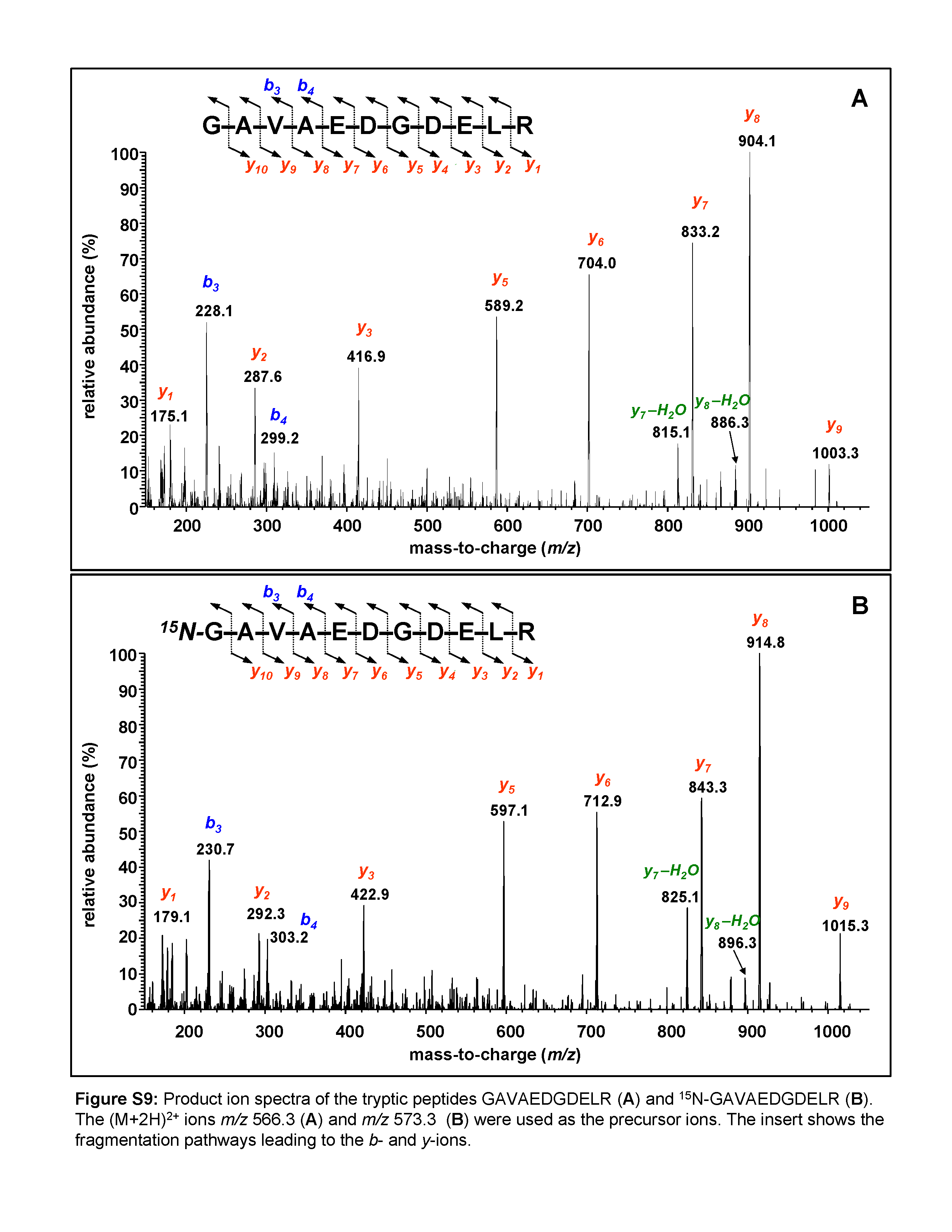

Supplement: Figure S9 — Product ion spectra of the tryptic peptides GAVAEDGDELR (A) and 15N-GAVAEDGDELR (B). The (M+2H)2+ ions m/z 566.3 (A) and m/z 573.3 (B) were used as the precursor ions. The insert shows the fragmentation pathways leading to the b- and y-ions. (TIFF) [file pone.0069894.s009.tiff]

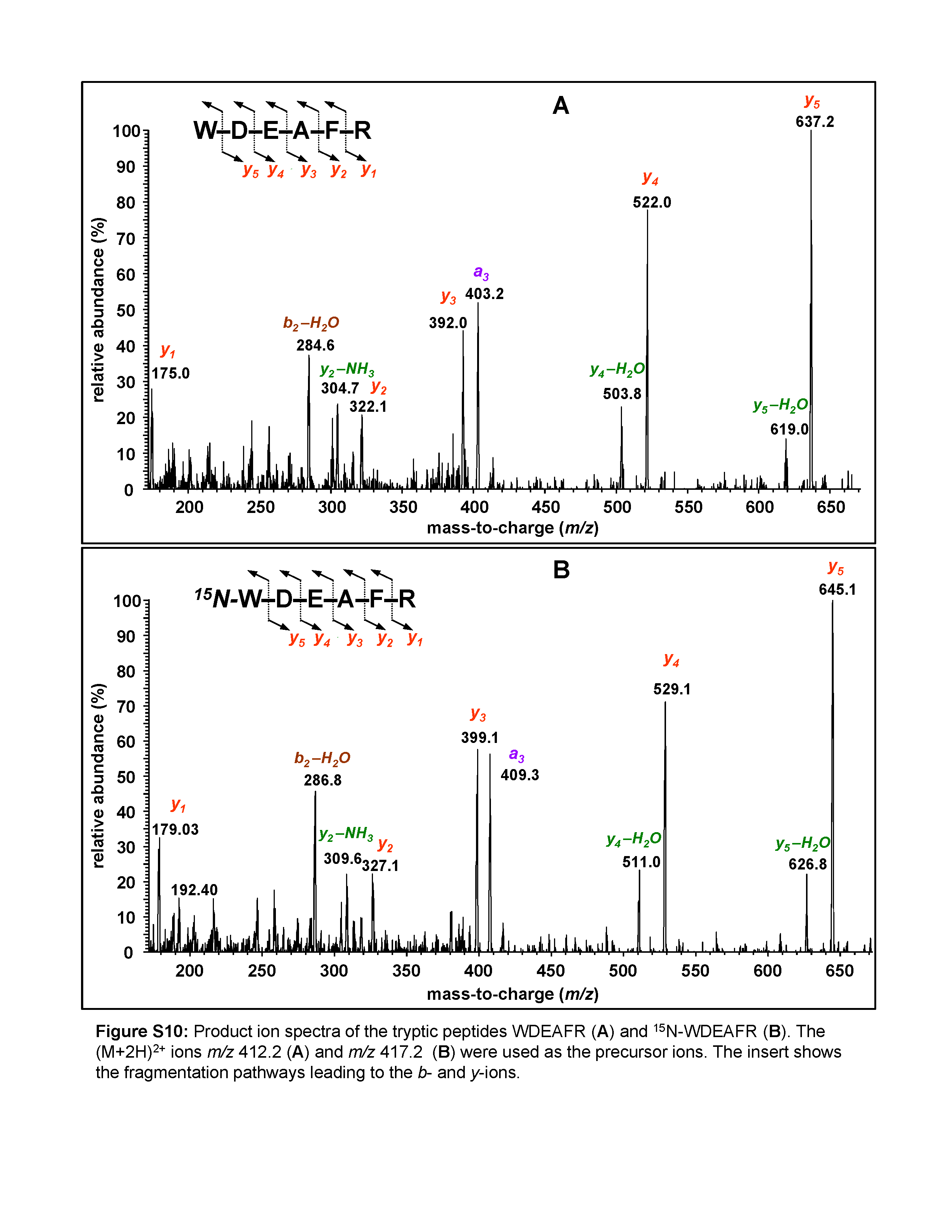

Supplement: Figure S10 — Product ion spectra of the tryptic peptides WDEAFR (A) and 15N-WDEAFR (B). The (M+2H)2+ ions m/z 412.2 (A) and m/z 417.2 (B) were used as the precursor ions. The insert shows the fragmentation pathways leading to the b- and y-ions. (TIFF) [file pone.0069894.s010.tiff]

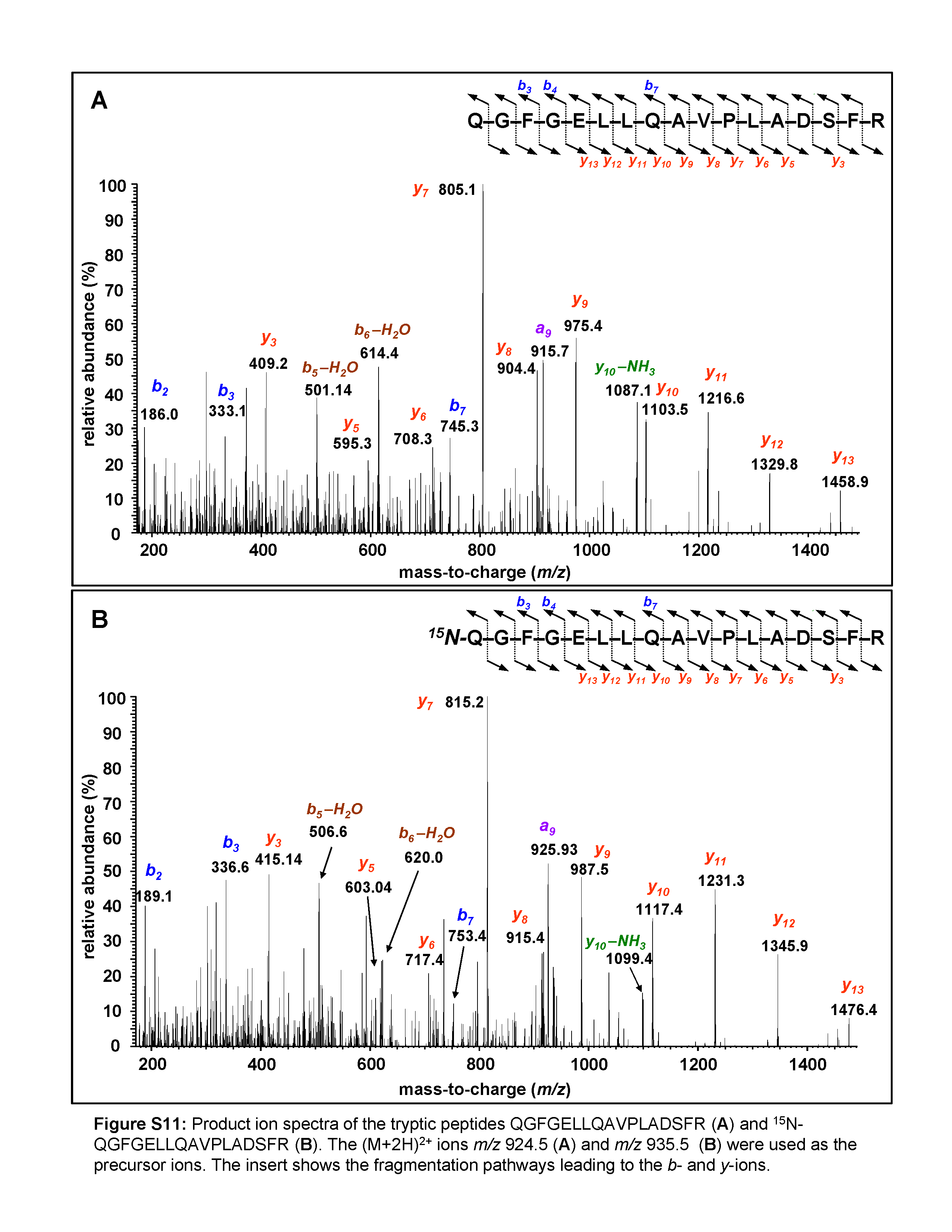

Supplement: Figure S11 — Product ion spectra of the tryptic peptides QGFGELLQAVPLADSFR (A) and 15N-QGFGELLQAVPLADSFR (B). The (M+2H)2+ ions m/z 924.5 (A) and m/z 935.5 (B) were used as the precursor ions. The insert shows the fragmentation pathways leading to the b- and y-ions. (TIFF) [file pone.0069894.s011.tiff]

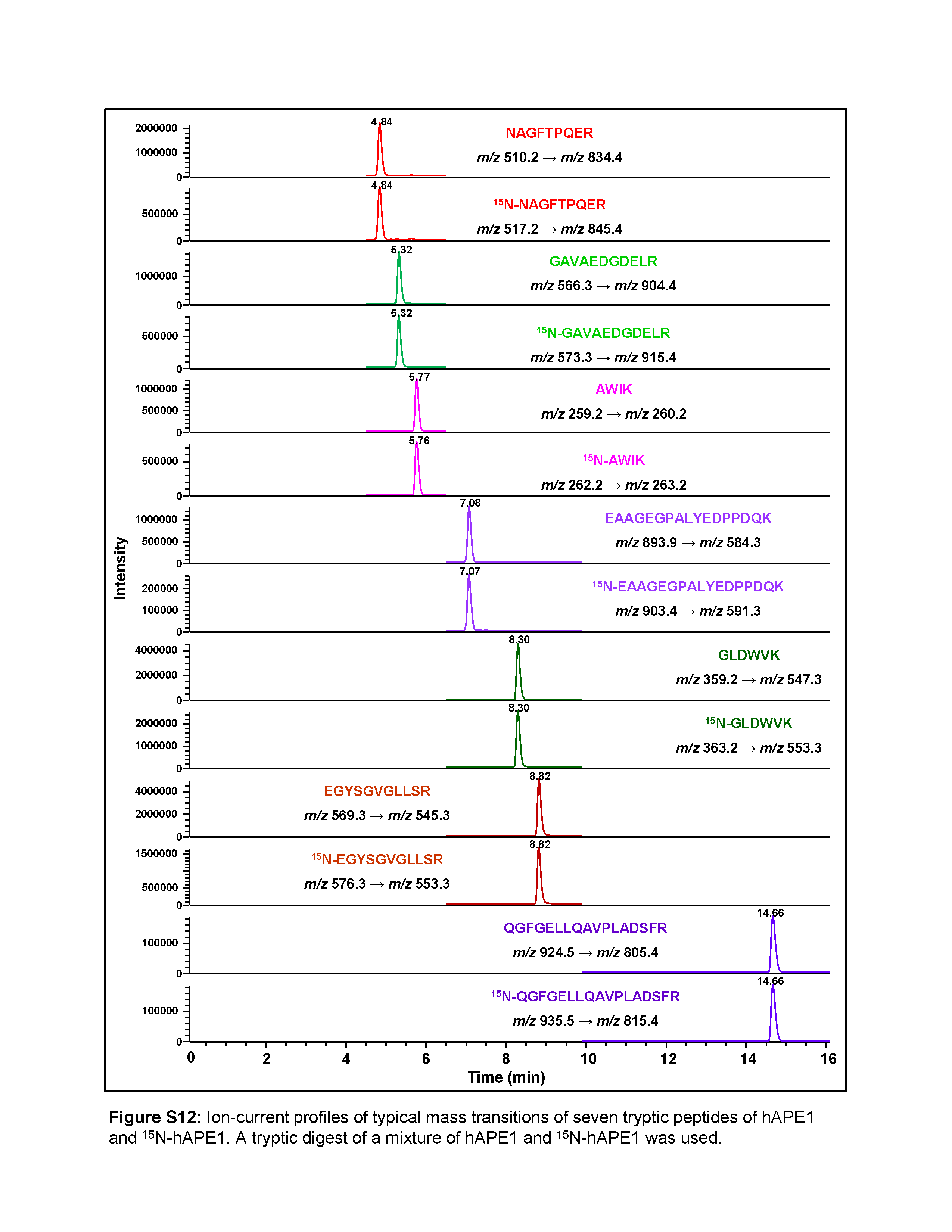

Supplement: Figure S12 — Ion-current profiles of typical mass transitions of seven tryptic peptides of hAPE1 and 15N-hAPE1. A tryptic digest of a mixture of hAPE1 and 15N-hAPE1 was used. (TIFF) [file pone.0069894.s012.tiff]

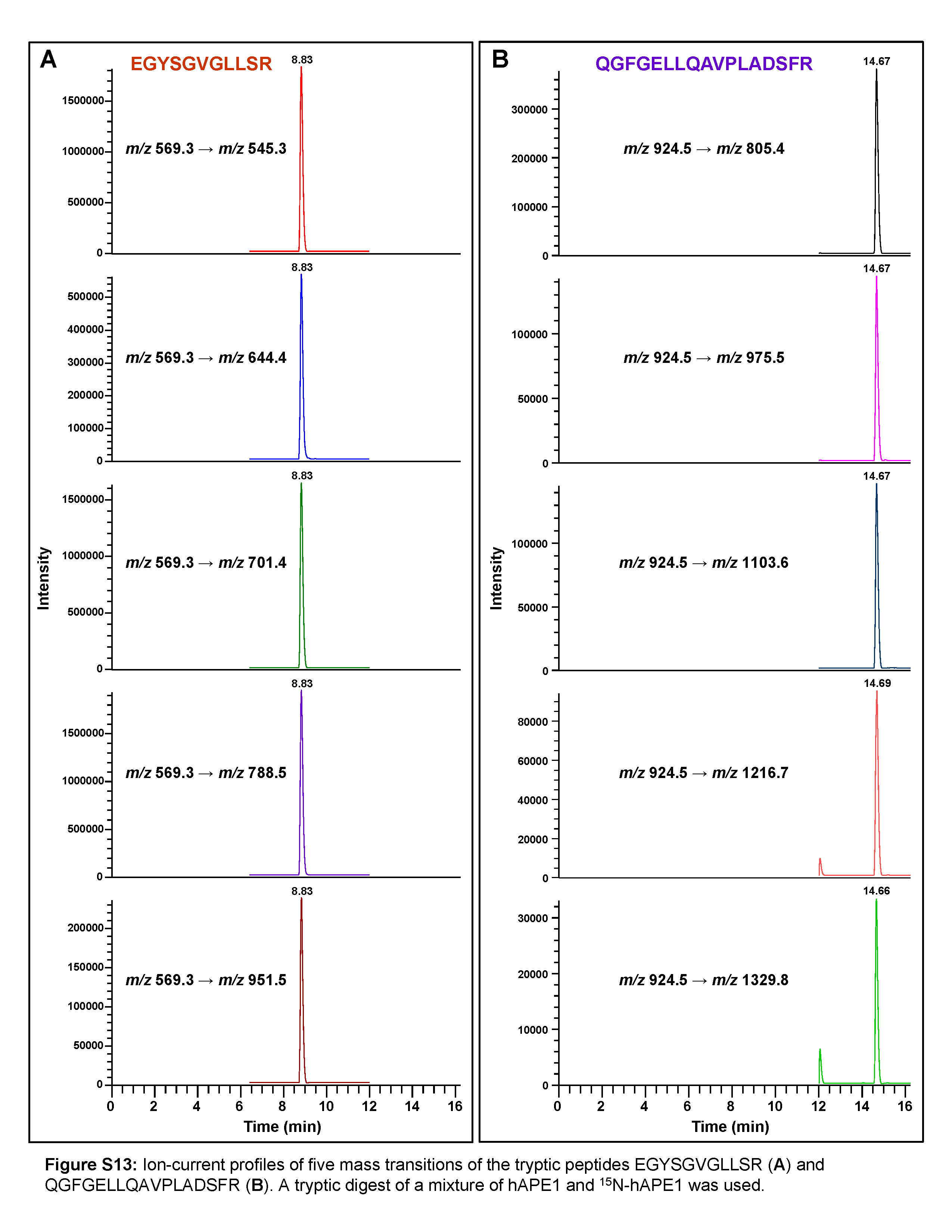

Supplement: Figure S13 — Ion-current profiles of five mass transitions of the tryptic peptides EGYSGVGLLSR (A) and QGFGELLQAVPLADSFR (B). A tryptic digest of a mixture of hAPE1 and 15N-hAPE1 was used. (TIFF) [file pone.0069894.s013.tiff]

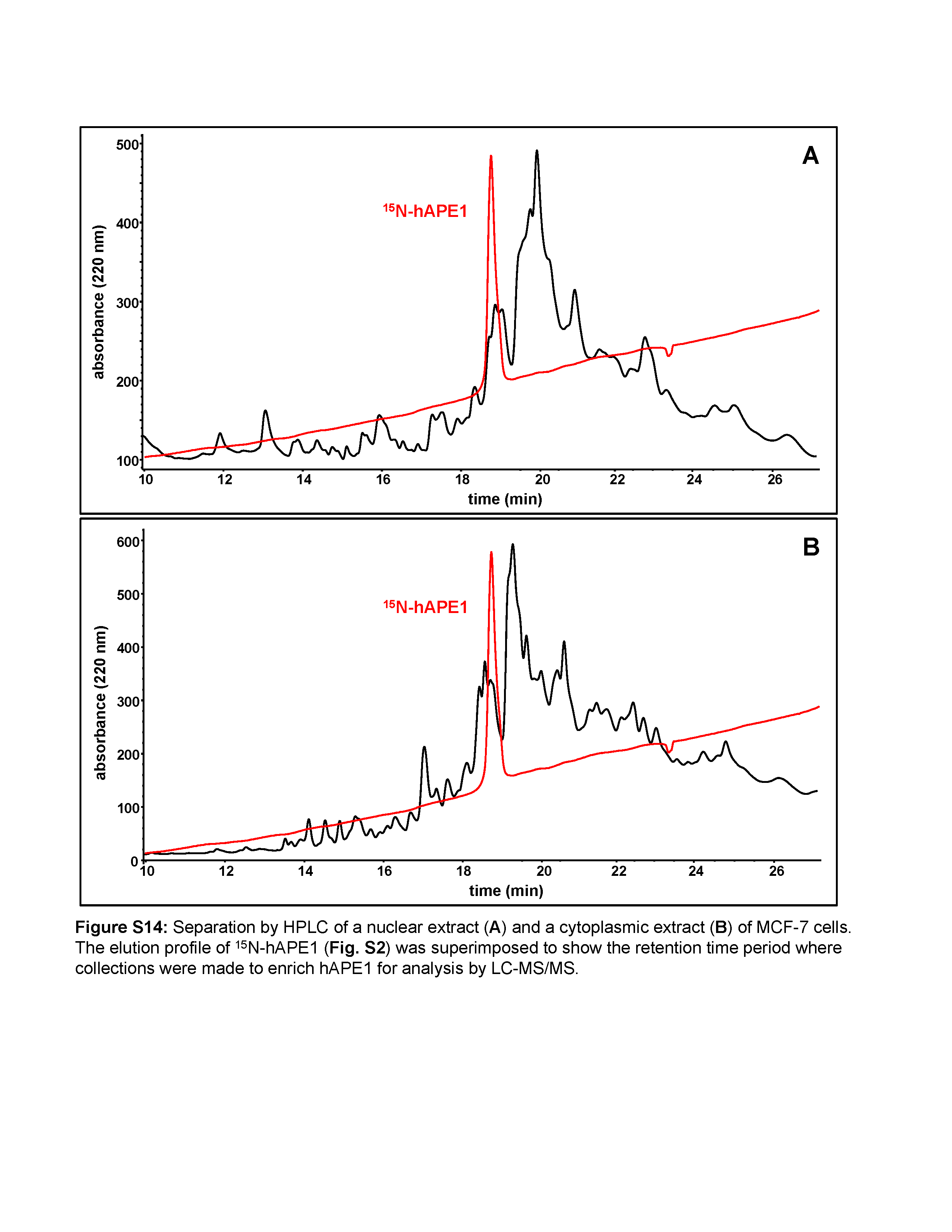

Supplement: Figure S14 — Separation by HPLC of a nuclear extract (A) and a cytoplasmic extract (B) of MCF-7 cells. The elution profile of 15N-hAPE1 (Fig. S2) was superimposed to show the retention time period where collections were made to enrich hAPE1 for analysis by LC-MS/MS. (TIFF) [file pone.0069894.s014.tiff]

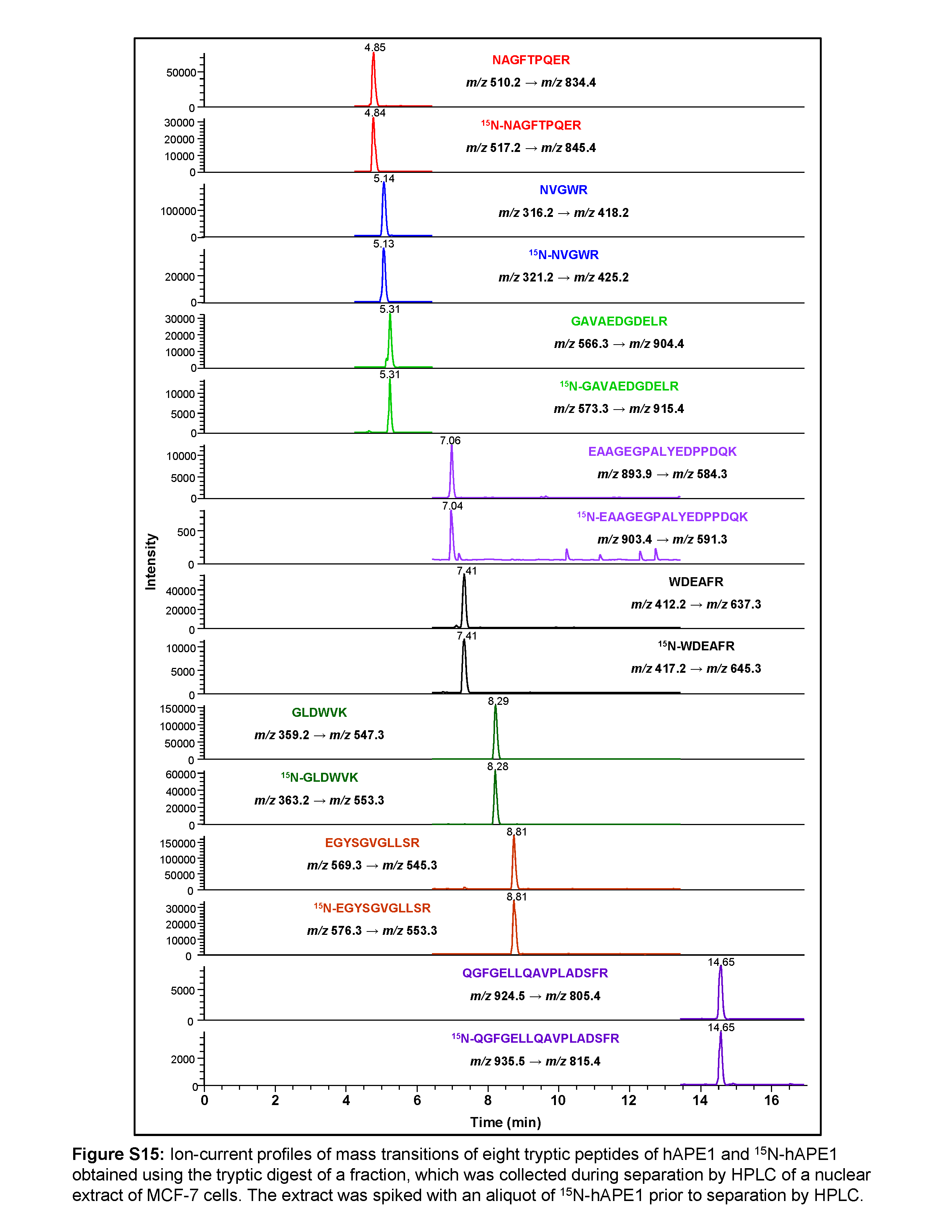

Supplement: Figure S15 — Ion-current profiles of mass transitions of eight tryptic peptides of hAPE1 and 15N-hAPE1 obtained using the tryptic digest of a fraction, which was collected during separation by HPLC of a nuclear extract of MCF-7 cells. The extract was spiked with an aliquot of 15N-hAPE1 prior to separation by HPLC. (TIFF) [file pone.0069894.s015.tiff]

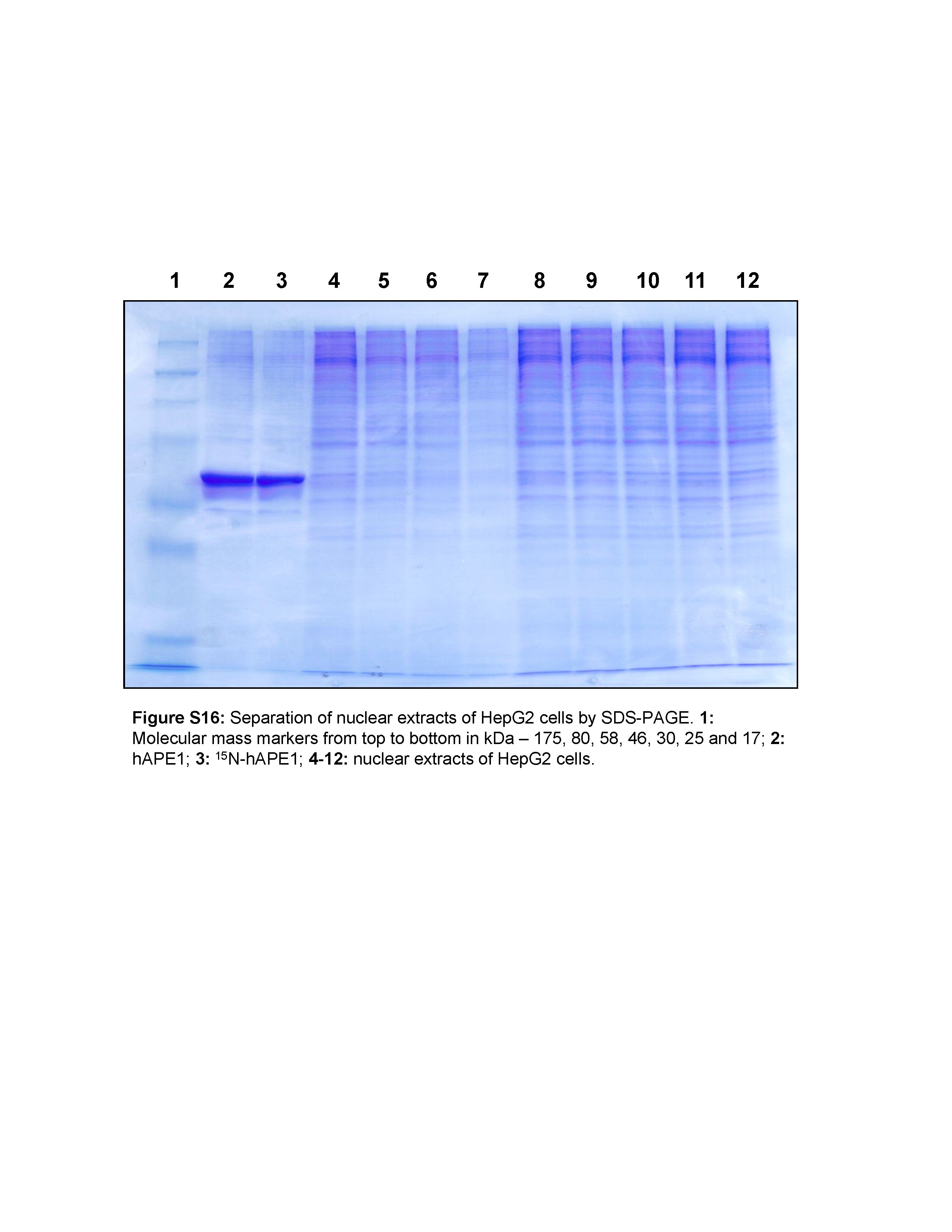

Supplement: Figure S16 — Separation of nuclear extracts of HepG2 cells by SDS-PAGE. 1: Molecular mass markers from top to bottom in kDa –175, 80, 58, 46, 30, 25 and 17; 2: hAPE1; 3∶ 15N-hAPE1; 4–12: nuclear extracts of HepG2 cells. (TIFF) [file pone.0069894.s016.tiff]

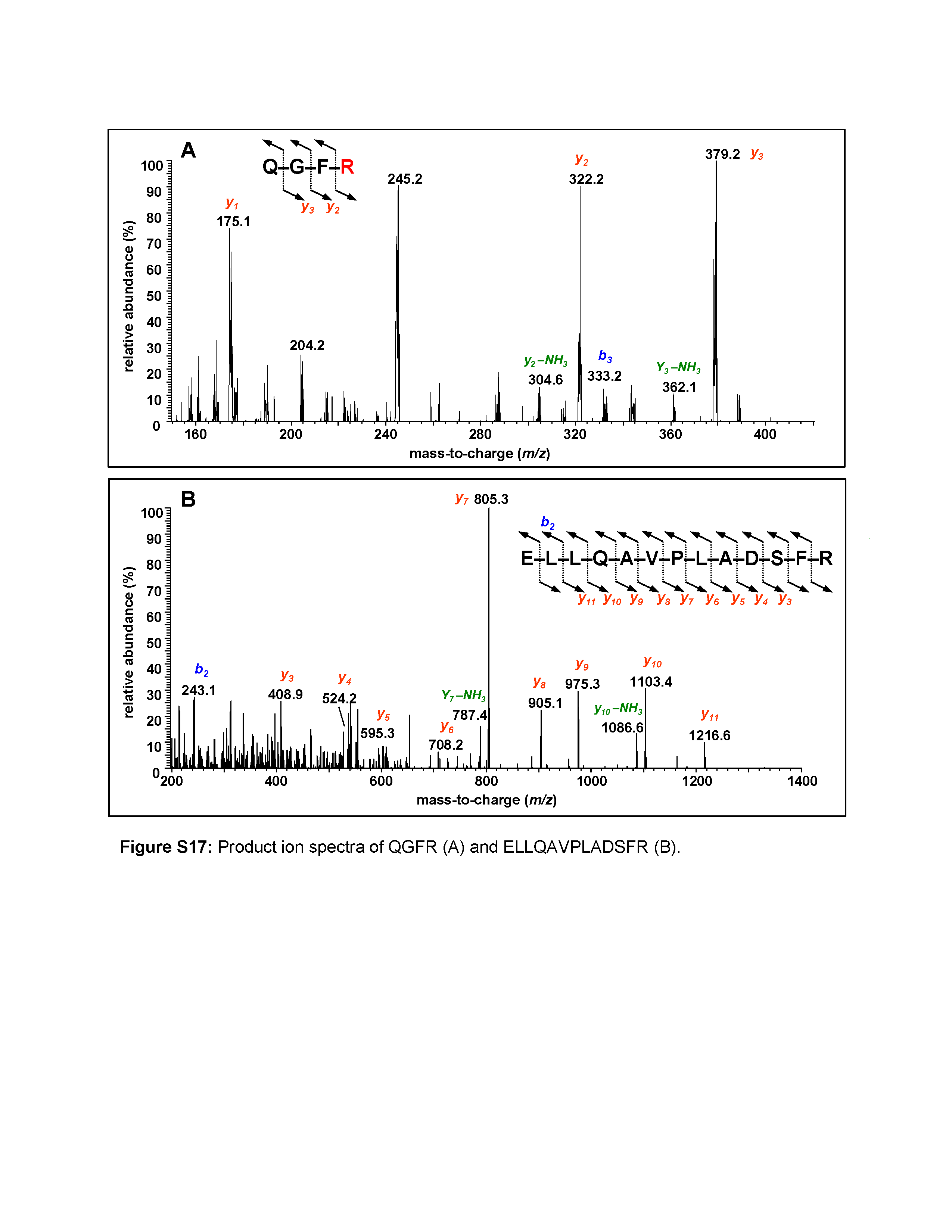

Supplement: Figure S17 — Product ion spectra of QGFR (A) and ELLQAVPLADSFR (B). (TIFF) [file pone.0069894.s017.tiff]

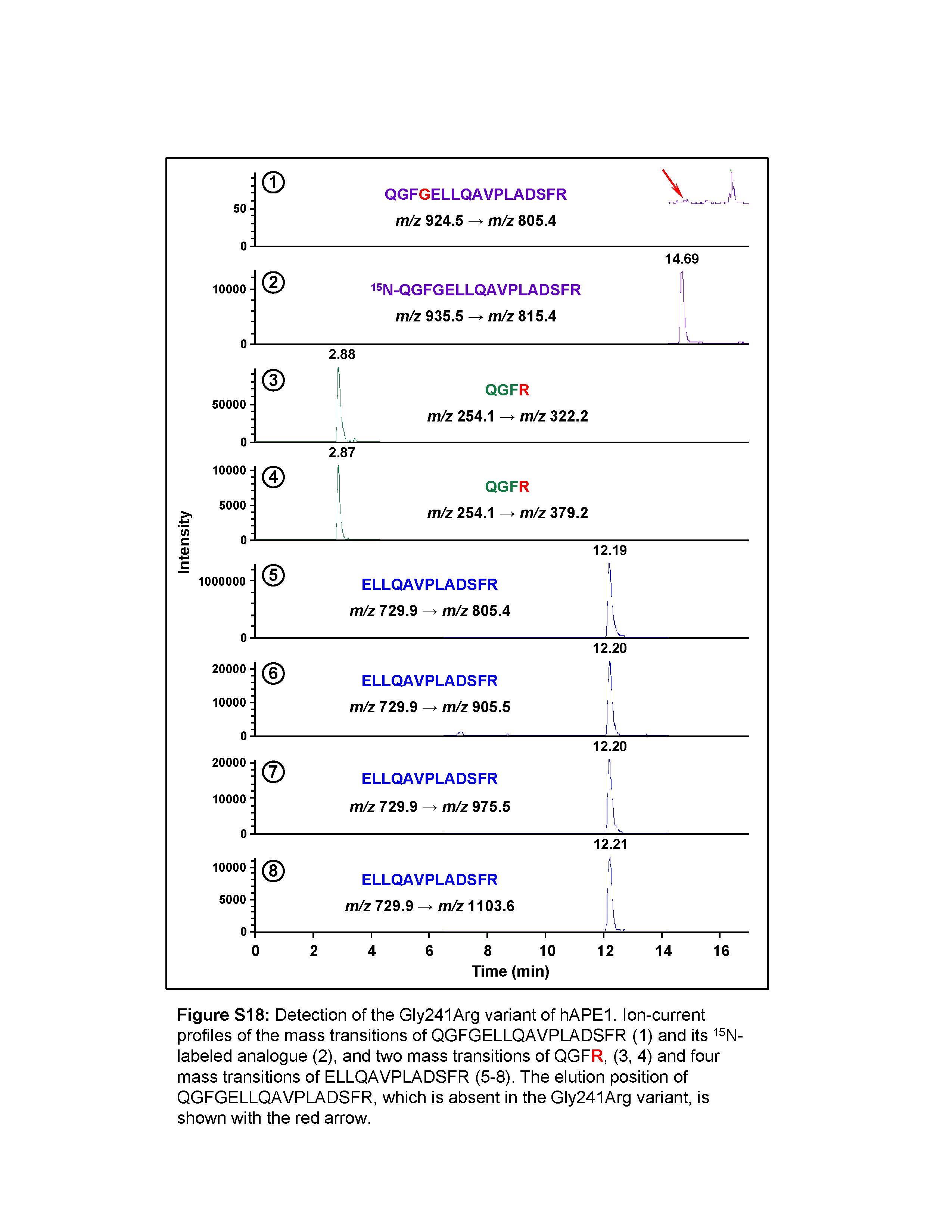

Supplement: Figure S18 — Detection of the Gly241Arg variant of hAPE1. Ion-current profiles of the mass transitions of QGFGELLQAVPLADSFR (1) and its 15N-labeled analogue (2), and two mass transitions of QGFR, (3, 4) and four mass transitions of ELLQAVPLADSFR (5–8). The elution position of QGFGELLQAVPLADSFR, which is absent in the Gly241Arg variant, is shown by the red arrow. (TIFF) [file pone.0069894.s018.tiff]

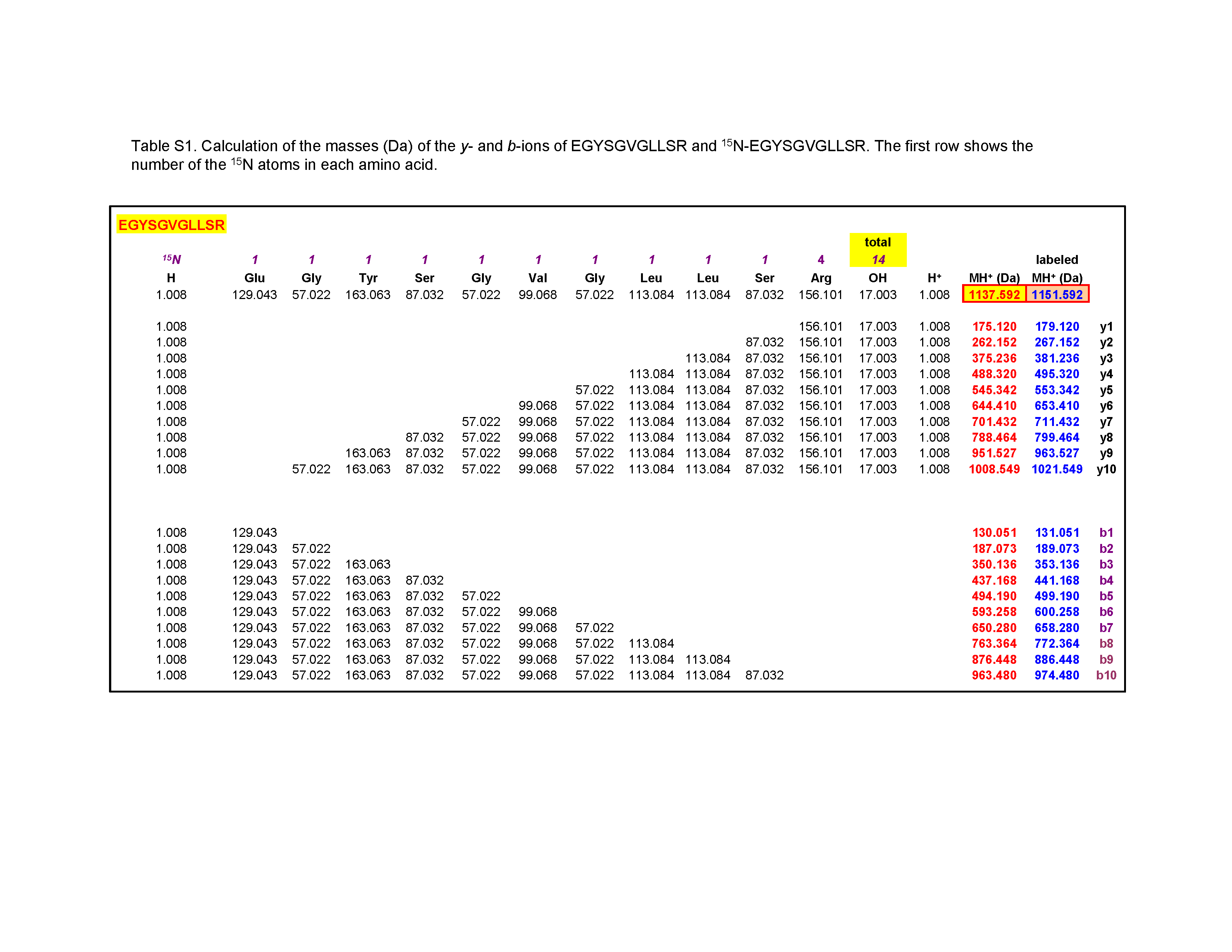

Supplement: Table S1 — Calculation of the masses (Da) of the y - and b -ions of EGYSGVGLLSR and 15N-EGYSGVGLLSR. The first row shows the number of the 15N atoms in each amino acid. (TIFF) [file pone.0069894.s019.tiff]

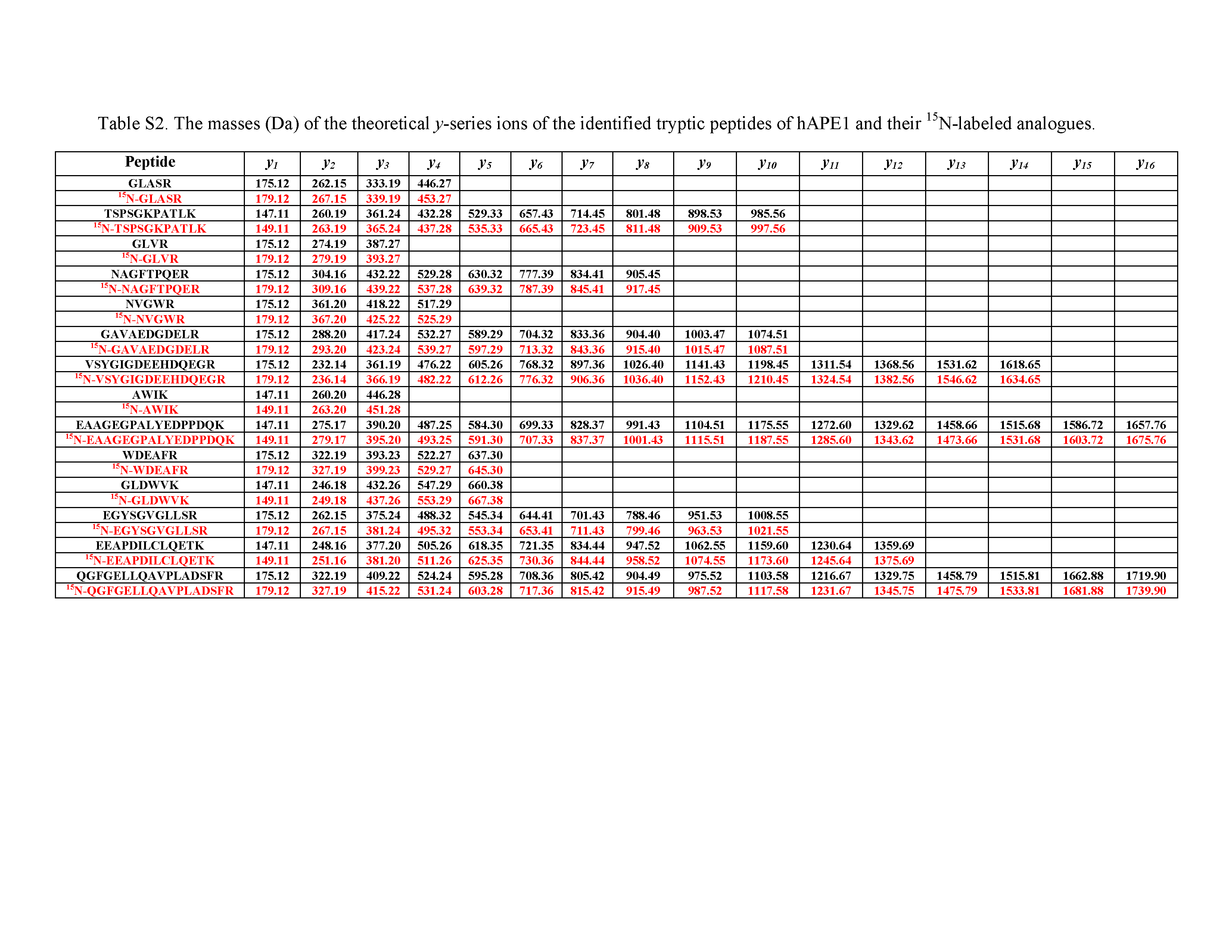

Supplement: Table S2 — The masses (Da) of the theoretical y -series ions of the identified tryptic peptides of hAPE1 and their 15N-labeled analogues. (TIFF) [file pone.0069894.s020.tiff]

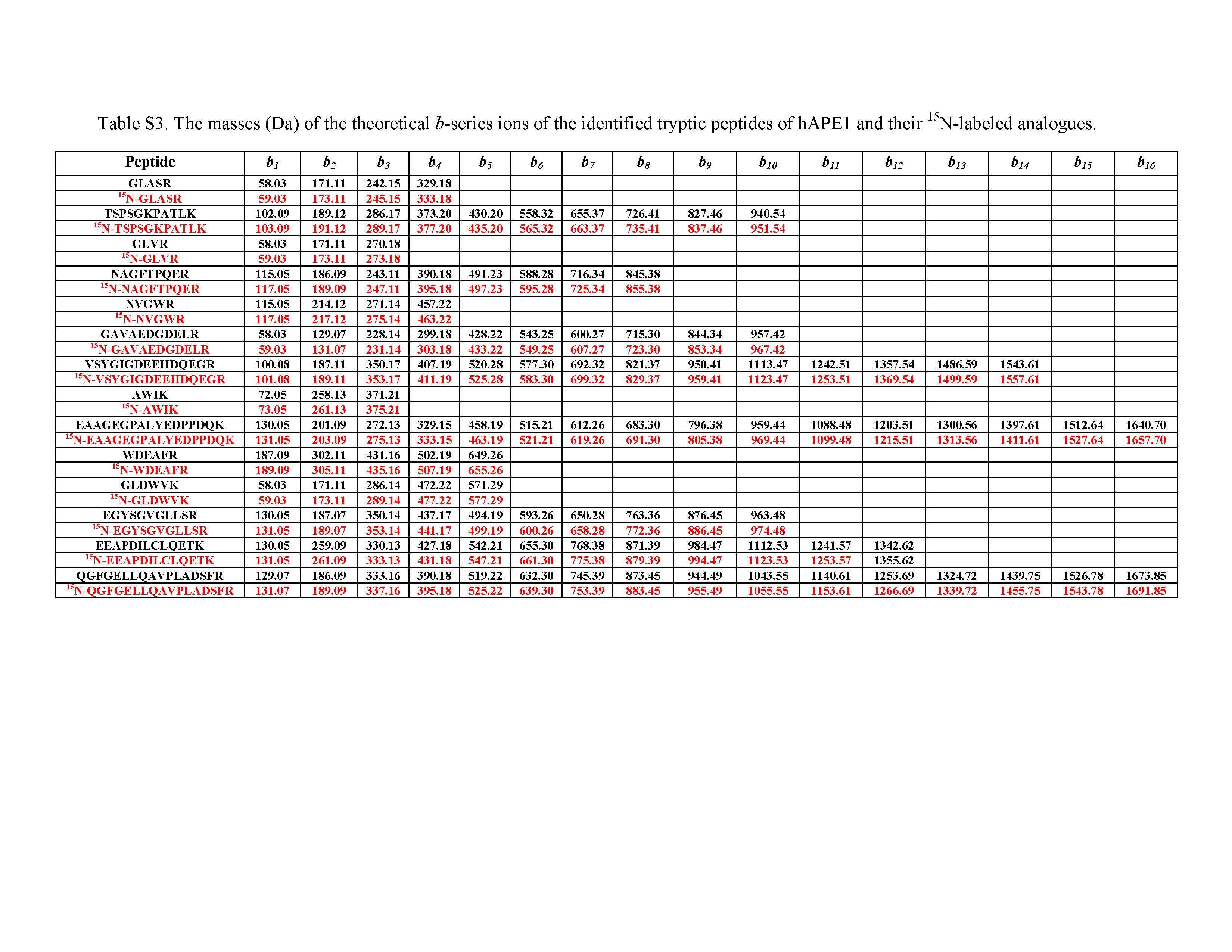

Supplement: Table S3 — The masses (Da) of the theoretical b -series ions of the identified tryptic peptides of hAPE1 and their 15N-labeled analogues. (TIF) [file pone.0069894.s021.tif]
